# Supplementary material for: SAMMY-seq reveals early alteration of heterochromatin and deregulation of bivalent genes in Hutchinson-Gilford Progeria Syndrome
Source: Nat Commun. 2020 Dec 8;11:6274. doi: 10.1038/s41467-020-20048-9 (PMC7722762; doi:10.1038/s41467-020-20048-9)
Supplement: Supplementary file 1 — Supplementary Information [file 41467_2020_20048_MOESM1_ESM.pdf]

Supplementary material for:

SAMMY-seq reveals early alteration of heterochromatin and deregulation of bivalent genes in Hutchinson-Gilford Progeria Syndrome

Endre Sebestyén[1§#], Fabrizia Marullo[2§], Federica Lucini[3‡], Cristiano Petrini[1‡], Andrea Bianchi[2,4], Sara Valsoni[4,5], Ilaria Olivieri[2], Laura Antonelli[5], Francesco Gregoretti[5], Gennaro Oliva[5], Francesco Ferrari[1,6]* and Chiara Lanzuolo[3,7]*

[1] IFOM, the FIRC Institute of Molecular Oncology, Milan, Italy
[2] Institute of Cell Biology and Neurobiology, National Research Council, Rome, Italy
[3] Istituto Nazionale Genetica Molecolare "Romeo ed Enrica Invernizzi", Milan, Italy
[4] IRCCS Santa Lucia Foundation, Rome, Italy
[5] Institute for High Performance Computing and Networking, National Research Council, Naples, Italy
[6] Institute of Molecular Genetics, National Research Council, Pavia, Italy
[7] Institute of Biomedical Technologies, National Research Council, Milan, Italy
[#] Current address: 1st Department of Pathology and Experimental Cancer Research, Semmelweis University, Budapest, Hungary

§ These authors contributed equally to this work and should be considered as co-first authors.
‡ These authors contributed equally to this work as second authors.
* These authors jointly supervised the work and should be considered as co-last authors.
Correspondence should be addressed to: chiara.lanzuolo@cnr.it; francesco.ferrari@ifom.eu

Table of Contents

Supplementary table 1 - Comparison with other high-throughput sequencing methods based on salt-extracted chromatin fractions 2
Supplementary table 2 - Sequenced reads 3
Supplementary table 3 - Characteristics of SAMMY-seq domains 4
Supplementary table 4 - SAMMY-seq domains overlap to LADs 5
Supplementary table 5 - Comparison with other high-throughput sequencing methods for genome-wide mapping of LADs and heterochromatin 6
Supplementary table 6 - Pairwise comparison of SAMMY-seq domains across control or progeria samples 7
Supplementary table 7 – Primer sequences........8
Supplementary figure 1 - Characteristics and reliability of SAMMY-seq libraries 9
Supplementary figure 2 - Detailed characteristics and genome-wide association of SAMMY-seq fractions. 11
Supplementary figure 3 - SAMMY-seq scale-down to small number of cells. 13
Supplementary figure 4 - Characteristics of control and progeria fibroblast cells 15
Supplementary figure 5 - SAMMY-seq profiles in early and late passage cells........ 16
Supplementary figure 6 - Additional analysis on H3K9me3 patterns. 17
Supplementary figure 7 - Expression analysis in control and progeria samples. 18
Supplementary figure 8 - Global H3K27me3 levels in HGPS are similar to controls........ 19
Supplementary figure 9 - Additional analysis on H3K27me3 patterns........ 20
Supplementary Methods........ 21
Supplementary References........23

	SAMMY-seq	Salt fractions profiling	HRS-seq
Genomic regions extracted	Heterochromatin	Euchromatin	Euchromatin
More fractions	YES	YES	NO
Permeabilisation	10% Triton, 10 min	1.5% NP40, 5 min	0.5% Triton 15 min
Enzyme for DNA digestion	Dnase, 1h	MNase, 10 min	NO
Low salt buffer	NO	YES, 1-2h	NO
High salt buffer	2M NaCl, 5 min	600mM NaCl, O/N	2M NaCl, 5 min
Wash step after salt extraction	YES	NO	YES
Post-extraction enzyme digestion	NO	NO	YES, StyI, 4h
Urea solubilisation	YES	NO	NO

Supplementary Table 1: Comparison with other high-throughput sequencing methods based on salt-extracted chromatin fractions. The table highlights the main characteristics and differences between SAMMY-seq and other genome-wide methods to study chromatin, based on different salt buffers for separating distinct chromatin fractions. These methods include "salt fractions profiling" [1] and HRS-seq [2].

Sample ID	Fraction	Number of reads
CTRL002	S2	78102212
CTRL002	S3	61341611
CTRL002	S4	55731571
CTRL004	S2	78683296
CTRL004	S3	60438514
CTRL004	S4	54864540
CTRL013	S2	59635985
CTRL013	S3	65360683
CTRL013	S4	85335120
HGPS167	S2	58983114
HGPS167	S3	73501327
HGPS167	S4	61985833
HGPS169	S2	79209359
HGPS169	S3	77731616
HGPS169	S4	92435639
HGPS188	S2	68067662
HGPS188	S3	104711260
HGPS188	S4	54037646

Supplementary Table 2: Sequenced reads. Total number of sequencing reads in each sample and fraction for the SAMMY-seq sequencing runs.

Sample ID	Comparison	Total length	Domain count	Domain average length	Autosome %
CTRL002	S3 vs S2	605.736895	273	2.218816465	21.06909824
CTRL002	S4 vs S2	616.186907	285	2.162059323	21.43257672
CTRL002	S4 vs S3	582.086898	276	2.1090105	20.24649008
CTRL004	S3 vs S2	374.950204	204	1.837991196	13.04173932
CTRL004	S4 vs S2	499.250205	205	2.435366854	17.36521533
CTRL004	S4 vs S3	477.000188	188	2.537235043	16.5913021
CTRL013	S3 vs S2	644.500193	193	3.339379238	22.41738615
CTRL013	S4 vs S2	510.136889	267	1.910625052	17.7438824
CTRL013	S4 vs S3	435.23686	238	1.828726303	15.13866538
HGPS167	S3 vs S2	178.500104	104	1.716347154	6.208695983
HGPS167	S4 vs S2	549.050231	231	2.376840827	19.09738923
HGPS167	S4 vs S3	487.250195	195	2.498718949	16.9478239
HGPS169	S3 vs S2	782.150186	186	4.205108527	27.20520946
HGPS169	S4 vs S2	465.712191	260	1.791200735	16.19867633
HGPS169	S4 vs S3	629.494655	176	3.576674176	21.89545467
HGPS188	S3 vs S2	540.336884	262	2.062354519	18.79431645
HGPS188	S4 vs S2	650.582792	197	3.302450721	22.6289547
HGPS188	S4 vs S3	693.286821	199	3.483853372	24.11431144

Supplementary Table 3: Characteristics of SAMMY-seq domains. The table contains the following columns. *Sample ID*: patient ID, *Comparison*: fractions used in the EDD run as the “IP” and “input” sample, *Total length*: total length of SAMMY-seq domains in megabase, *Domain count*: number of SAMMY-seq domains, *Domain average length*: average length of SAMMY-seq domains in megabase, *Autosome %*: percentage of autosomes covered by SAMMY-seq domains.

Sample ID	Comparison	Lamin A/C overlap JI	Lamin A/C overlap %	Lamin B1 overlap JI	Lamin B1 overlap %
CTRL002	S3 vs S2	0.541625	72.06768	0.460908	74.30458
CTRL002	S4 vs S2	0.545483	71.77053	0.462623	73.76663
CTRL002	S4 vs S3	0.524959	72.08383	0.455923	75.47672
CTRL004	S3 vs S2	0.381417	74.50327	0.307011	74.91666
CTRL004	S4 vs S2	0.536448	79.44917	0.401284	75.72359
CTRL004	S4 vs S3	0.556347	83.46960	0.398524	77.53669
CTRL013	S3 vs S2	0.606142	75.02715	0.466940	72.37393
CTRL013	S4 vs S2	0.438633	68.54847	0.393770	73.71361
CTRL013	S4 vs S3	0.276098	53.29121	0.268582	61.10281

Supplementary Table 4: SAMMY-seq domains overlap to LADs. The table contains the following columns. *Sample ID*: patient ID, *Comparison*: fractions used in the EDD run as the “IP” and “input” sample, *Lamin A/C overlap JI*: Jaccard index of the SAMMY-seq domain – Lamin A/C LAD comparison as calculated by bedtools, *Lamin A/C overlap %*: percentage of SAMMY-seq domains overlapping with Lamin A/C LADs, *Lamin B1 overlap JI*: Jaccard index of the SAMMY-seq domain – Lamin B1 LAD comparison as calculated by bedtools, *Lamin B1 overlap %*: percentage of SAMMY-seq domains overlapping with Lamin B1 LADs. Note that for Lamin A/C and Lamin B1 we used a comprehensive set of published ChIP-seq profiles[3-7]. In order to define a consensus set of ChIP-seq enrichment domains we considered regions detected as enriched by the EDD algorithm in at least 2 of the published Lamin datasets that were reanalyzed (see Results and Methods sections).

	DamID	ChIP-seq H3K9me3	ChIP-seq Lamin A or B	Gradient -seq	Protect-seq	SAMMY -seq
Can be applied on primary cells and tissue		**YES**	**YES**	**YES**	**YES**	**YES**
Can be applied on single cell	**YES**					
Can be applied on small number of cells (10K)	**YES**	(*)		**YES**		**YES**
Avoid the use of antibody	**YES**			**YES**	**YES**	**YES**
Avoid the use of chemical modification of chromatin	**YES**	(**)				**YES**
Avoid transfection of constructs		**YES**	**YES**	**YES**	**YES**	**YES**
**Time to perform the protocol**	**(***) 2 days**	**2 days**	**2 days**	**(****) 2 days**	**4 hours**	**3 hours**

Supplementary Table 5: Comparison with other high-throughput sequencing methods for genome-wide mapping of LADs and heterochromatin. The table highlights the main characteristics and differences between SAMMY-seq and other genome-wide methods to study LADs and heterochromatin including DamID[8], ChIP-seq (targeting either H3K9me3 or Lamin proteins), gradient-seq [9,10] and protect-seq [11].

Notes in the table

(*) Possible with non-standard protocol (ChIPmentation) [12]

(**) Possible with a non-standard protocol (N-ChIP) [13]

(***) Construct cloning, production and transfection not included

(****) With sucrose gradient protocol version [9]

Sample ID 1	Sample ID 2	Comparison	Overlap JI	Overlap number
CTRL002	CTRL004	S3 vs S2	0.536316	177
CTRL002	CTRL013	S3 vs S2	0.585113	188
CTRL004	CTRL013	S3 vs S2	0.39536	137
HGPS167	HGPS169	S3 vs S2	0.190102	84
HGPS167	HGPS188	S3 vs S2	0.282751	93
HGPS169	HGPS188	S3 vs S2	0.482547	186
CTRL002	CTRL004	S4 vs S3	0.686345	187
CTRL002	CTRL013	S4 vs S3	0.46086	178
CTRL004	CTRL013	S4 vs S3	0.370164	127
HGPS167	HGPS169	S4 vs S3	0.172627	107
HGPS167	HGPS188	S4 vs S3	0.121931	100
HGPS169	HGPS188	S4 vs S3	0.717687	172
CTRL002	CTRL004	S4 vs S2	0.707946	200
CTRL002	CTRL013	S4 vs S2	0.695666	242
CTRL004	CTRL013	S4 vs S2	0.614278	190
HGPS167	HGPS169	S4 vs S2	0.168257	76
HGPS167	HGPS188	S4 vs S2	0.0748601	85
HGPS169	HGPS188	S4 vs S2	0.0823542	71

Supplementary Table 6. Pairwise comparison of SAMMY-seq domains across control or progeria samples. The table contains the following columns. *Sample ID 1*: patient ID for comparison, *Sample ID 2*: patient ID for comparison, *Comparison*: fractions used in the EDD run as the "IP" and "input" sample, *Overlap JI*: Jaccard index of the SAMMY-seq domain overlaps as calculated by bedtools, *Overlap number*: number of overlapping SAMMY-seq domains.

Name	Sequence	H3K27me3	H3K9me3
GAPDH-FW	ATCCAAGCGTGTAAGGGTCC	Negative	
GAPDH-REV	GACTGAGATTGGCCCGATGG	Negative	
CD4-FW	TGATCTCAGCCTCTCGTTCC	Positive	
CD4-REV	CTTCGAGACCTTTGCCTCC	Positive	
H3K4me1chr18-FW	AGACGCTGACCACAACTGG		Negative
H3K4me1chr18-REV	TGGATGCCCAGCATGGTTG		Negative
H3K9me3chr16-FW	AGCTGTGGTATCCTCACCG		Positive
H3K9me3chr16-REV	GACTGCCTTACAGAGACGC		Positive

Supplementary Table 7. Primer sequences.
Details for primers used for q-PCR experiments for ChIP-seq validation. Regions expected to be amplified are indicated as positive while regions not expected to be amplified are indicated as negative. FW: forward; REV: reverse.

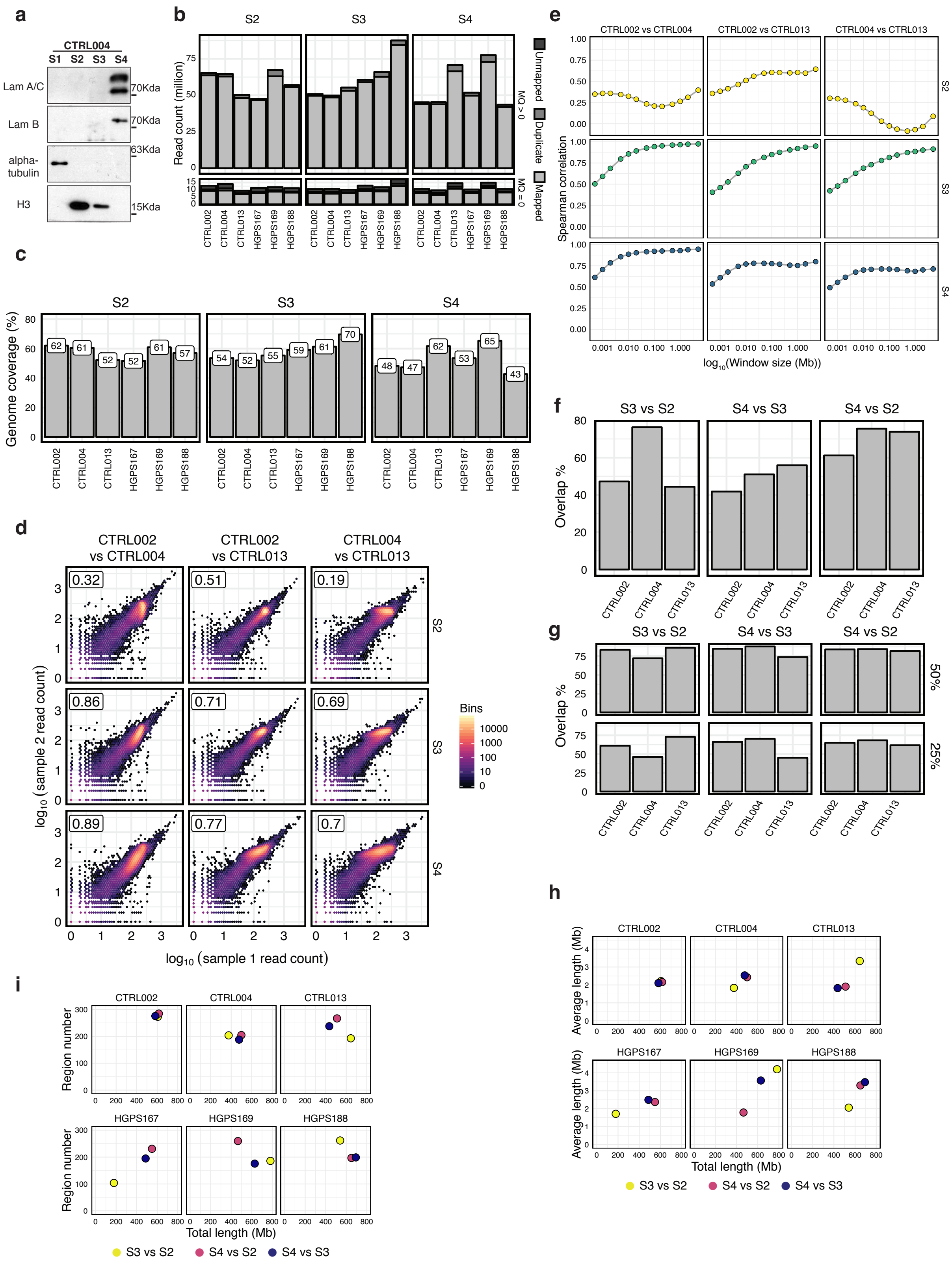

Supplementary Figure 1 - Characteristics and reliability of SAMMY-seq libraries. a, Representative western blot for chromatin fractionation experiments of CTRL004. Sequential extractions were performed to isolate soluble proteins (S1-fraction), DNase-sensitive chromatin (S2-fraction), DNase-resistant chromatin (S3-fraction), and the most compact and inaccessible chromatin (S4-fraction). Equal amounts of each fraction were hybridized with indicated antibodies. **b**, Total read counts for each SAMMY-seq sample. The stacked bar plot shows mapped, unmapped, and PCR duplicate reads with bwa mapping quality equal to zero (MQ = 0, that were

discarded from subsequent analyses) or larger than zero (MQ > 0). The average number of sequencing reads in the different fractions were 72 million (S2), 62 million (S3) and 65 (S4) million. **c**, Percentage of the human genome (hg38-noalt build) covered by at least one read in each SAMMY-seq sample. On average 58% (S2), 54% (S3) and 52% (S4) of the genome was covered by at least one read. The labels on each bar report the associated exact value. **d**, Scatter plots comparing read counts over 10Kb bins in the S2, S3 and S4 fractions (rows) across three pairwise comparisons of the control samples (columns). The color gradient represents the number of genomic bins with the same (x,y) values. Spearman correlation values are reported in the top left corner of each subpanel. **e**, Spearman correlation (y-axis) of read counts between control samples pairs (labels on top of the plots) computed over different bin sizes (x-axis, in log scale) in the S2, S3 and S4 fractions (labels on the right-hand side). **f**, Percentage of SAMMY-seq domains (S3 vs S2, S4 vs S3 and S4 vs S2) conserved across all 3 control samples (computed as percent over per sample total SAMMY-seq domains size). **g**, Percentage of SAMMY-seq domains (S3 vs S2, S4 vs S3 and S4 vs S2) detected after 50% or 25% down sampling of sequencing reads (computed as percent over per sample total SAMMY-seq domains size detected with 100% of sequencing reads). **h**, Average (y-axis) and total (x-axis) size of SAMMY-seq domains (S3 vs S2, S4 vs S3 and S4 vs S2) for each control and HGPS sample **i**, Number (y-axis) and total size (x-axis) of SAMMY-seq domains (S3 vs S2, S4 vs S3 and S4 vs S2) for each control and HGPS sample.

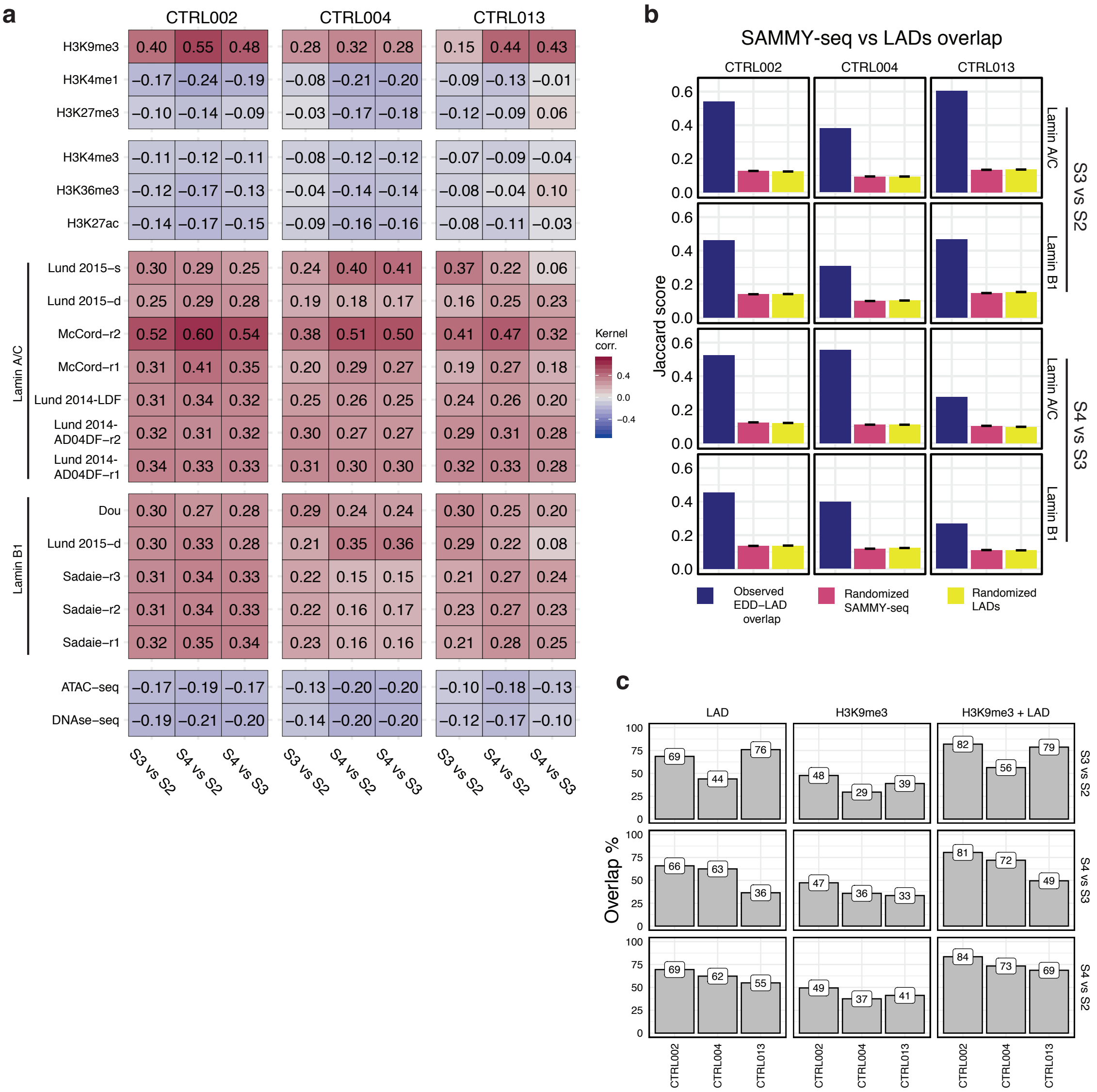

Supplementary Figure 2 - Detailed characteristics and genome-wide association of SAMMY-seq fractions. a, Genome-wide kernel correlation calculated by StereoGene, between SAMMY-seq fraction comparisons in individual control samples against ChIP-seq and other chromatin marks. These include: ATAC-seq, DNase-seq, Lamin A/C and Lamin B1, H3K27me3, H3K4me1, H3K9me3, H3K36me3, H3K27ac and H3K4me3. For a more detailed analysis of correlation with Lamin association, we considered multiple public datasets for Lamin A/C and Lamin B1. ChIP-seq, as indicated in the individual row labels: multiple replicates from Lund *et al.* 2015[5], McCord et al. [6], Lund et al. 2014[4], Dou *et al.*[3] and Sadaie *et al.*[7]. For each control samples (CTRL002, CTRL004, CTRL013 – labels on top) and each SAMMY-seq fractions comparison (S3 vs S2; S4 vs S3; S4 vs S2 – labels at the bottom) the correlation is high for closed chromatin marks and lower for open chromatin marks. It must be noted that a subselection of these same correlation values (for S4 vs S2 comparison) is reported in Figure 2b, along with the average correlation values for Lamin A/C and Lamin B1. **b**, Overlap (Jaccard Index - JI) of 3 biologically independent control samples SAMMY-seq domains (S3 vs S2 or S4 vs S3, labels on the right hand side) with LADs (Lamin A/C or Lamin B1 ChIP-seq enrichment domains consensus set). In order to define a consensus set of ChIP-seq enrichment domains for either Lamin A/C or Lamin B1 LADs we considered regions detected as enriched by the EDD algorithm in at least 2 of the datasets reanalised in panel **"a"** of this same figure for either of these proteins (see also Results and Methods sections). The observed JI (blue bar) is compared to median JI across 10,000 randomizations of SAMMY-seq domains (pink bar) or LADs (yellow bar) positions along the genome to compute empirical p-values for the probability of the observed JI being larger than the random expectation. All one tail

empirical p-values were significant ($p < 0.0001$). The whiskers over the randomized values show the +/-2 SEM interval. **c**, Percentage of LAD, H3K9me3 or LAD+H3K9me3 regions detected by the S4 vs S2 SAMMY-seq domains. LADs were defined based on Lamin A/C ChIP-seq enrichment domains consensus set (as described above), while H3K9me3 regions were originating from the Roadmap Epigenomics skin fibroblasts (E055) sample[14].

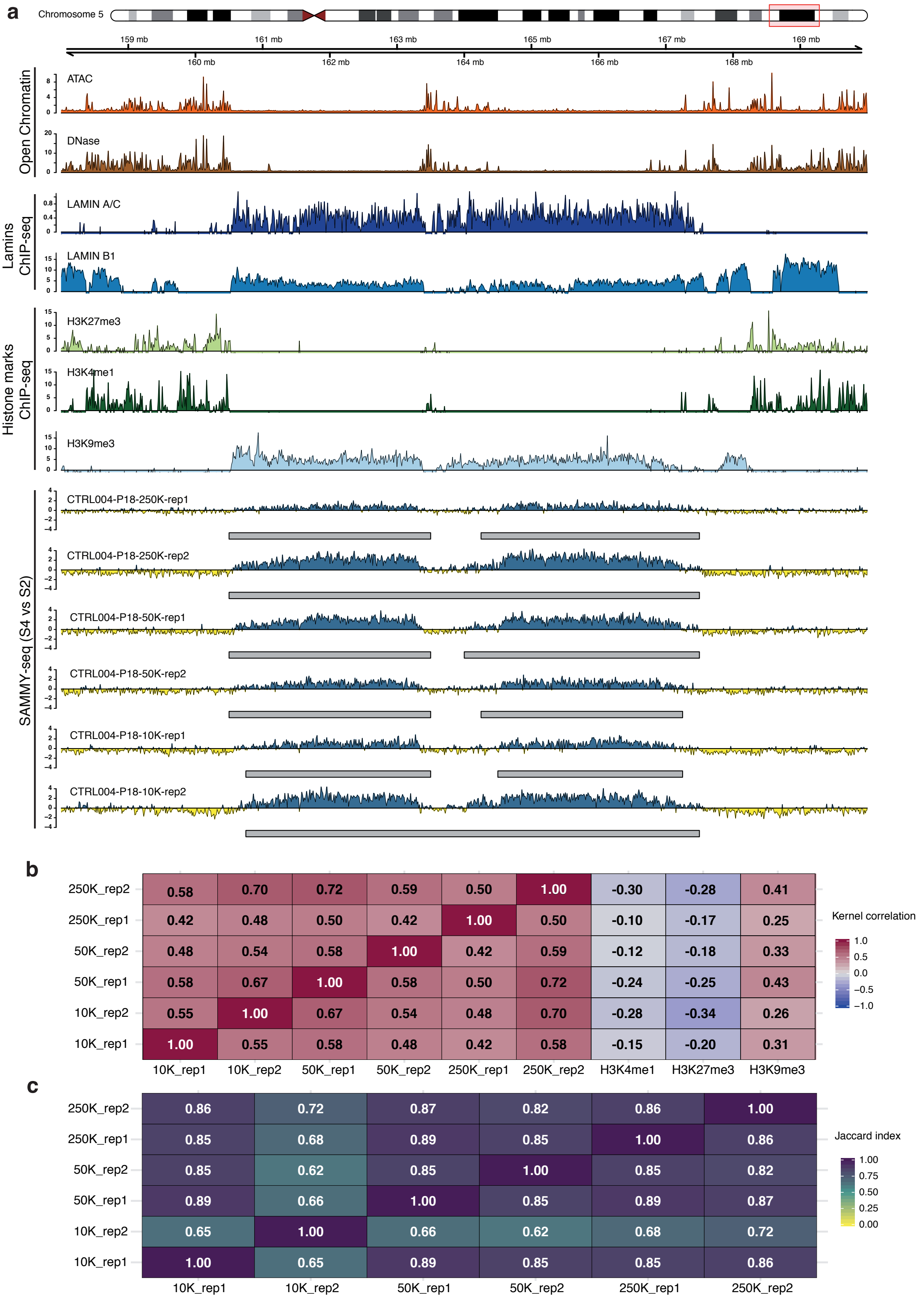

Supplementary Figure 3 - SAMMY-seq scale-down to small number of cells. a, Visualization of SAMMY-seq enrichment profiles along with multiple chromatin marks on a representative region (8Mb region in chr5:158000000-170000000). From top to bottom: tracks for open chromatin (ATAC-seq and DNase-seq); association to lamina (Lamin A/C and Lamin B1 ChIP-seq); ChIP-seq for histone marks associated to PcG regulation (H3K27me3) and active chromatin (H3K4me1); ChIP-seq for histone marks associated to heterochromatin (H3K9me3); SAMMY-seq enrichment signal (S4 vs S2) in a representative control fibroblast sample (CTRL004) at passage 18, starting from 250K, 50K or 10K cells, each of them in 2 replicates (rep1 and rep2) as indicated in the label

for each genomic track. Grey boxes under each SAMMY-seq track show the SAMMY-seq domains. See Methods for details of each type of signal processing. This is the same region and epigenomics tracks as reported in Figure 2a, to facilitate the comparison with the new SAMMY-seq profiles obtained in the scale-down experiment. **b**, Genome-wide kernel correlation (StereoGene) between all pairs of SAMMY-seq enrichment signal profiles for the same control fibroblast samples shown in panel “**a**”. StereoGene kernel correlation is also reported for the comparison between the same SAMMY-seq samples and ChIP-seq profiles for histone marks H3K4me1, H3K27me3 and H3K9me3. **c**, Pairwise overlap (Jaccard Index) of SAMMY-seq domains (S4 vs S2) identified in the SAMMY-seq samples for the same control fibroblast samples shown in panel “**a**”.

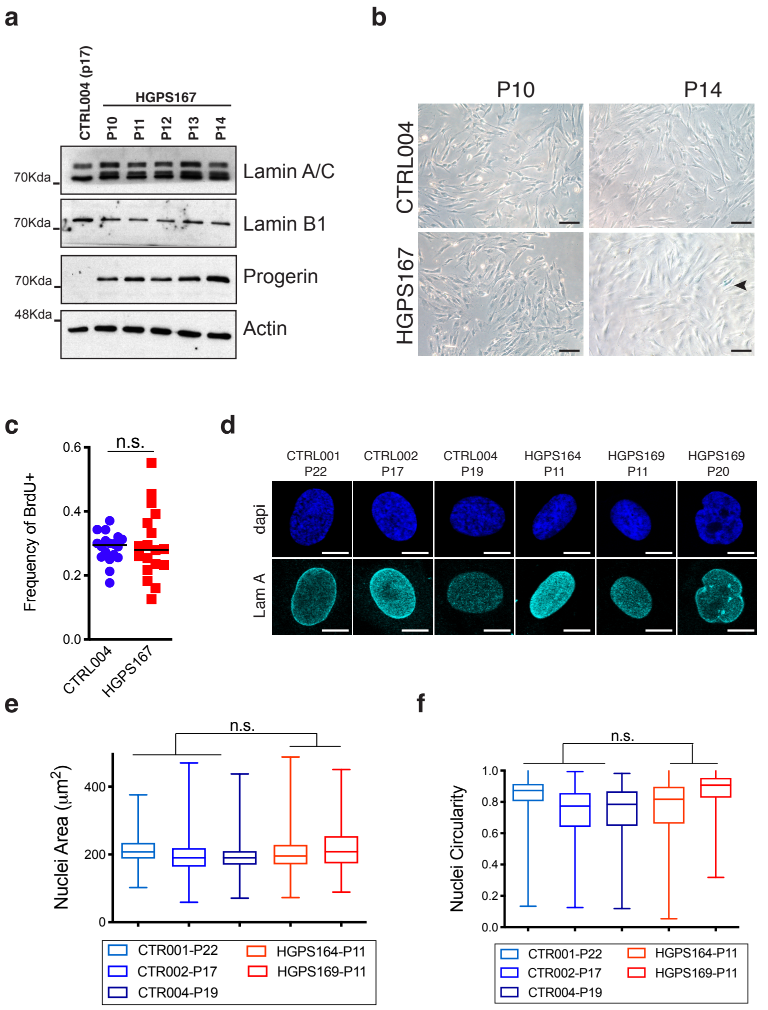

Supplementary Figure 4 - Characteristics of control and progeria fibroblast cells. a, Representative western blots for total extract from CTRL and HGPS fibroblasts at indicated passages (P10-14) hybridized with indicated antibodies. The experiment was done twice on biological independent samples. **b**, Representative fields of SA-β-gal activity in CTRL and HGPS fibroblasts at indicated passages (P10-14). Scale bar, 50 μm. The experiment was performed on one biological sample. **c**, Scatter dot plot reporting the proliferation rate in control vs HGPS (P10) fibroblasts estimated as percentage of BrdU positive cells with respect to the total number of nuclei in each field (total analysed nuclei > 550). The plot reports averages of two biological independent experiments, horizontal black line is the median. Each dot represents the proliferation rate of a confocal imaging field. **d**, Representative images of Lamin A/C/DAPI immunofluorescence analysis on control or HGPS fibroblasts at various passages (indicated with P) selected from 3 or 2, respectively control and HGPS, independent biological replicates. Scale bars: 10 μm. **e**, **f**, Quantification of nuclei area (panel e) and circularity (panel f) of CTRL and HGPS fibroblasts. Horizontal lines within the boxes represent the medians, upper and lower bounds of the boxes represent quartiles Q3 (75th percentile) and Q1 (25th percentile), respectively, and the whiskers min to max. Number of analyzed nuclei over 3 independent biological replicates: CTRL001-P22: 660; CTRL002-P17: 1253; CTRL004-P19: 885. ; Number of analyzed nuclei over 2 independent biological replicates: HGPS164-P11: 990; HGPS169-P11: 312. Comparisons were tested using a two-tail t-test in (c) and one-way nested ANOVA (test on F statistic) in (e, f). n.s.: not significant.

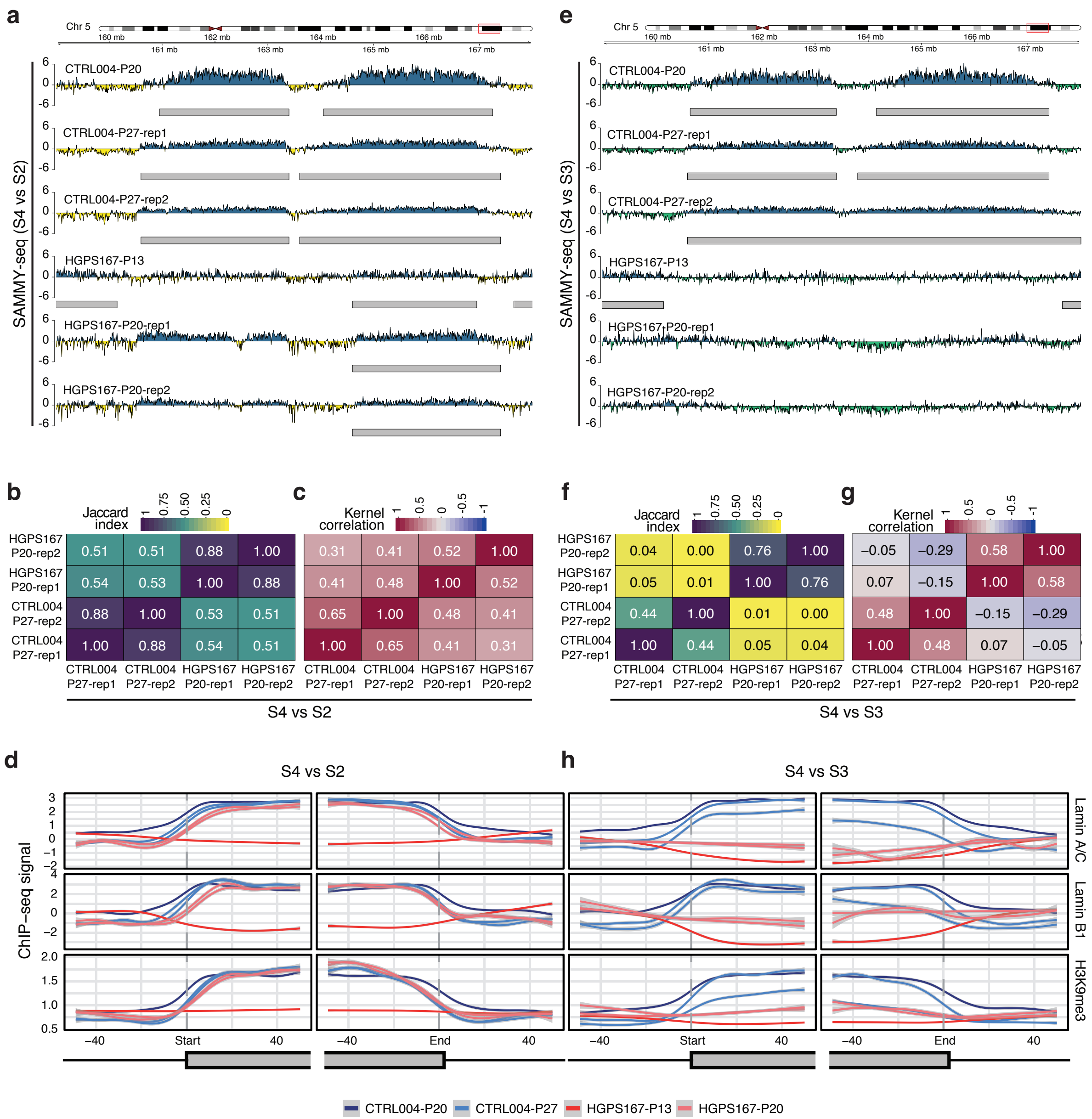

Supplementary Figure 5 - SAMMY-seq profiles in early and late passage cells. **a**, Genomic tracks for SAMMY-seq profiles (S4 vs S2 fractions comparison) for a representative region (8Mb region in chr5:159000000-168000000) for healthy human fibroblasts (CTRL004) and HGPS primary fibroblasts (HGPS167) at indicated early and late passages. Rep1 and 2 indicate two biological replicates. Grey boxes under each SAMMY-seq track show the SAMMY-seq domains. **b**, Pairwise overlap (Jaccard index) of the SAMMY-seq domains called in the S4 vs S2 fractions comparison. **c**, Genome-wide kernel correlation (StereoGene) of the SAMMY-seq profiles for S4 vs S2 fractions comparison. **d**, Smoothed average ChIP-seq enrichment signal for chromatin marks around SAMMY-seq domain borders. ChIP-seq signal for Lamin A/C (upper row), Lamin B1 (middle row) and H3K9me3 (bottom row) is reported around the SAMMY-seq domains called in the S4 vs S2 fractions comparison for either early or late passage control (blue lines – colour legend) or HGPS (red lines – colour legend) samples, using a +/-50 bins window (10Kb bin size) centred on the start or end domain border positions (vertical dashed grey line). The smoothed average is obtained by GAM (see methods) and the grey shaded area under each line shows the 95% confidence interval for the fitted GAM. **e**, same as panel (a) but for S4 vs S3 fractions comparison. **f**, **g**, same as panel (b) and (c), respectively, but for S4 vs S3 fractions comparison. **h**, same as panel (d) but for S4 vs S3 fractions comparison.

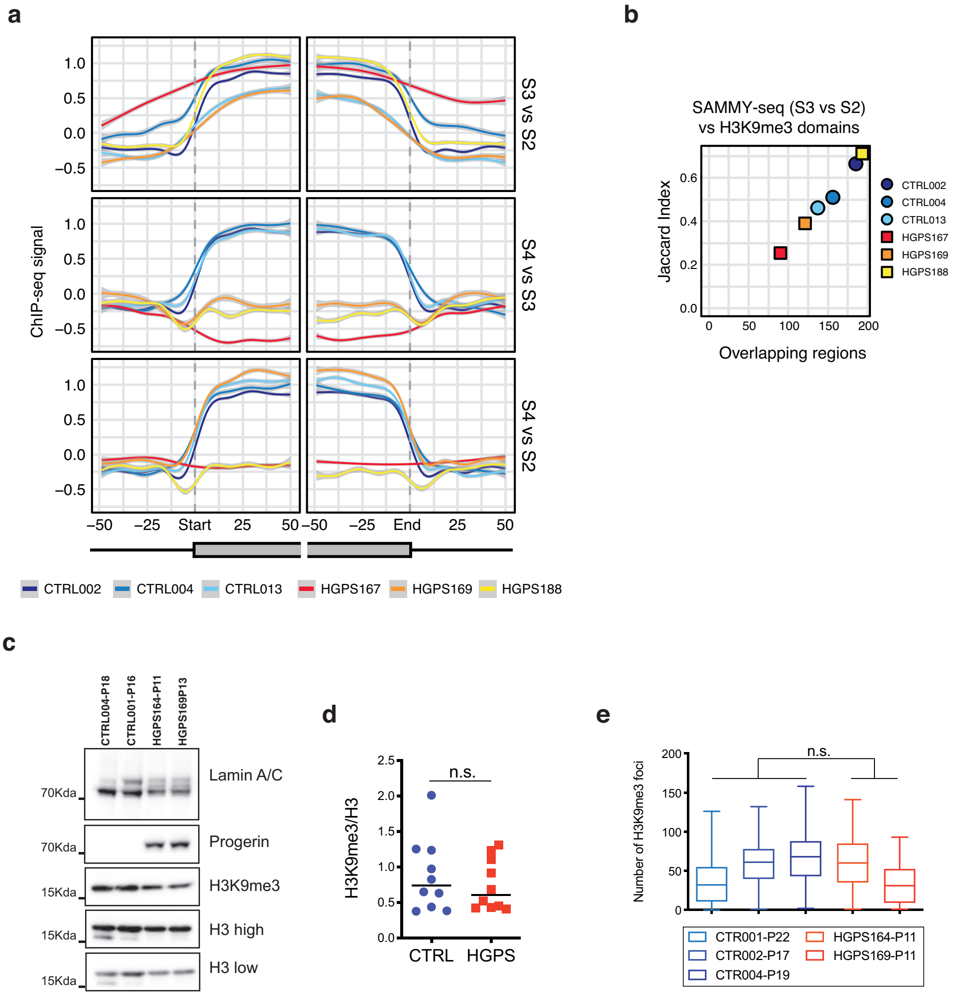

Supplementary Figure 6 - Additional analysis on H3K9me3 patterns. a, Smoothed average ChIP-seq enrichment signal for H3K9me3 is reported around the SAMMY-seq domain borders start (left side plots) or end (right side plots) for domains detected in each control (black lines) and progeria (red lines) sample. H3K9me3 ChIP-seq was obtained for each sample individually. Results for each set of SAMMY-seq enrichment domains are reported (S3 vs S2 top, S4 vs S3 middle, S4 vs S2 bottom) using a +/-50 bins window (10Kb bin size) centered on the start or end domain border positions (vertical dashed grey line). The smoothed average is obtained by GAM (see methods) and the grey shaded area under each line shows the 95% confidence interval for the fitted GAM. **b**, Overlap of H3K9me3 enriched domains and SAMMY-seq domains (S3 vs S2) for control or HGPS samples (JI on y-axis, number of overlapping regions on x-axis). **c**, Representative western blots for total extract from control or HGPS fibroblasts at indicated early passages (P) hybridized with indicated antibodies. Histone H3 was used as loading controls for H3K9me3 quantification. **d**, Scatter dot plot reporting the quantifications of bands in (c) with H3K9me3 levels normalized on H3. Data points were generated from an average of at least 10 biological independent samples, horizontal black line is the median. **e**, Quantification of number per nucleus of H3K9me3 foci of CTRL and HGPS fibroblasts. Horizontal lines within the boxes represent the medians, upper and lower bounds of the boxes represent quartiles Q3 (75th percentile) and Q1 (25th percentile), respectively, and the whiskers min to max. Number of analyzed nuclei over 3 independent biological replicates: CTRL001-P22: 242; CTRL002-P17: 649; CTRL004-P19: 518. Number of analyzed nuclei over 2 independent biological replicates: HGPS164-P11: 550; HGPS169-P11: 65. Comparisons were tested using two-tail t-test in (d). We applied one-way nested ANOVA (test on F statistic) and generalised linear model with Poisson distribution (two-tail test on beta coefficient) in (e) and they were both not significant. n.s.: not significant.

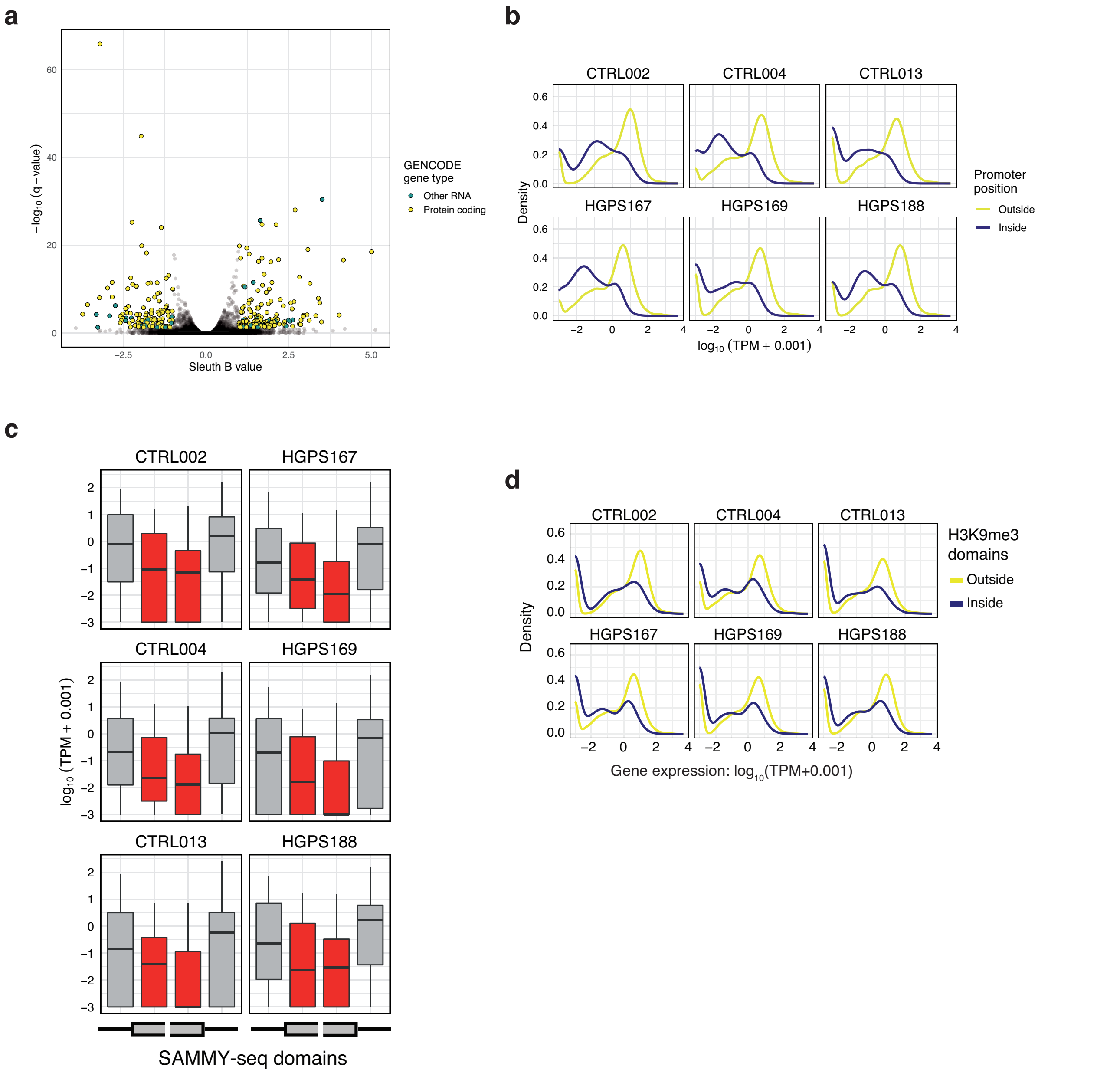

Supplementary Figure 7 – Expression analysis in control and progeria samples. a, Volcano plot of gene level differential expression. The x axis shows the sleuth b value, while the y axis shows -$\log_{10}$(q-value) of genes. Genes highlighted in green or yellow show significant changes ($|b| > 1$ and q-value < 0.05). The yellow color refers to protein coding genes based on GENCODE v27 annotation, green refers to all other types of non-protein coding genes. **b**, Normalized gene expression distribution for protein coding genes separated based on their promoter being inside or outside of the consensus SAMMY-seq domains (S4 vs S2) of all controls. Genes with promoters outside the domains have higher expression (two-sided Wilcoxon rank sum test p-values < 0.01). **c**, Boxplot of normalized gene expression distribution for protein coding genes flanking consensus SAMMY-seq domain borders (S4 vs S2) of all 3 biologically independent controls. The gray boxplots show genes located in the outer 500Kb flanking regions (upstream of start or downstream of end), the red boxplots show genes in the inner 500Kb flanking regions (all two-sided Wilcoxon rank sum test p-values < 0.01). For each box the median is marked as a horizontal line, the lower and upper hinges mark the first and third quartiles (the 25th and 75th percentiles), the whishers extend up to 1.5 IQR from the hinge. **d**, Gene expression distribution for protein coding genes separated based on their position being inside or outside of the H3K9me3 peaks in each sample. All samples show a significant difference in expression (two-sided Wilcoxon rank sum test p-values < 0.01).

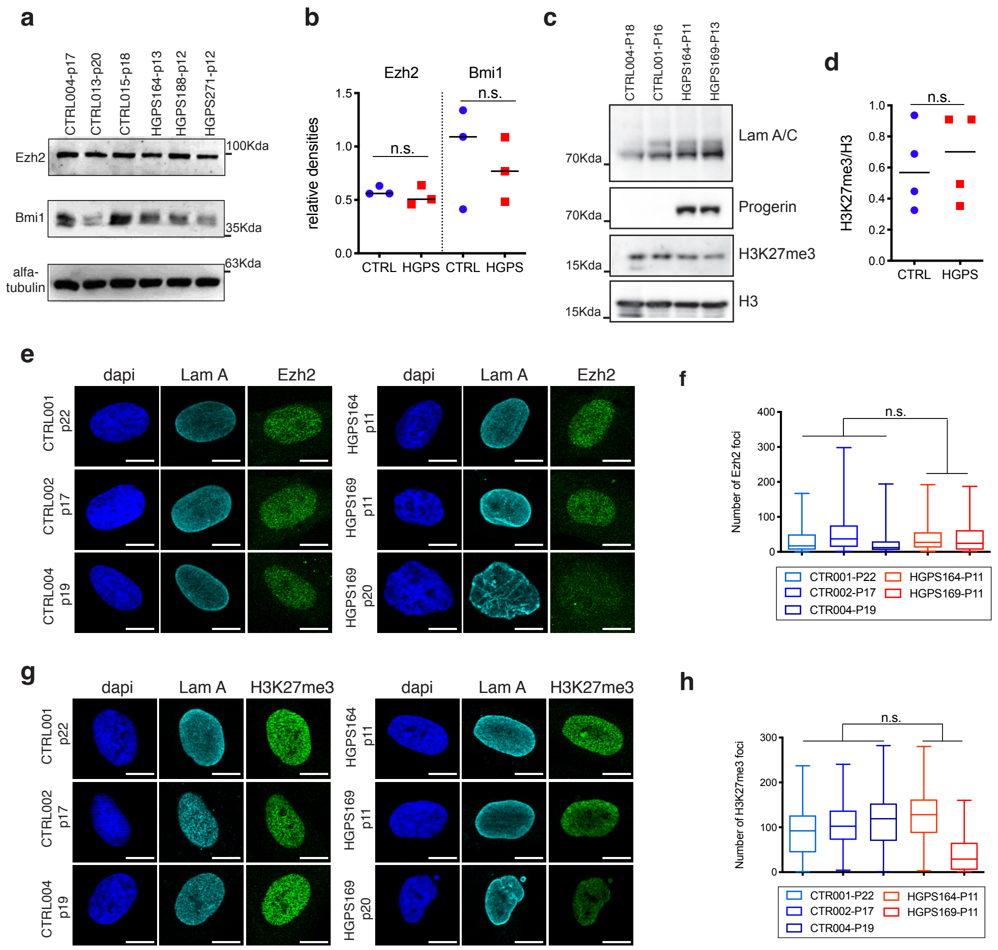

Supplementary Figure 8 – Global H3K27me3 levels in HGPS are similar to controls. a, Representative western blots for total extract from control or HGPS fibroblasts at the indicated early passages (P) hybridized with indicated antibodies. Alpha-tubulin was used as loading control. **b**, Scatter dot plot reporting the quantifications of of Ezh2 and Bmi1 levels normalized on Alpha-tubulin. Data points were generated from an average of at least 3 biological independent samples, horizontal black line is the median. **c**, Representative western blots for total extract from control or HGPS fibroblasts at the indicated early passages hybridized with indicated antibodies. Histone H3 was used as loading controls for H3K27me3 quantification. **d**, Scatter dot plot reporting the quantifications of H3K27me3 levels normalized on H3. Data points were generated from an average of at least 4 biological independent samples, horizontal black line is the median. **e**, Representative images of Ezh2/Lamin A immunofluorescence analysis on control or HGPS fibroblasts at indicated passages (P11-22). Scale bars: 10 µm. **f** Quantification of number per nucleus of Ezh2 foci of CTRL and HGPS fibroblasts. Horizontal lines within the boxes represent the medians, upper and lower bounds of the boxes represent quartiles Q3 (75th percentile) and Q1 (25th percentile), respectively, and the whiskers min to max. Number of analyzed nuclei: CTRL001-P22: 225; CTRL002-P17: 265; CTRL004-P19: 202; HGPS164-P11: 188; HGPS169-P11: 118. **g**, Representative images of H3K27me3/Lamin A immunofluorescence analysis on control or HGPS fibroblasts at indicated passages (P11-22). Scale bars: 10 µm. **h**, Quantification of number per nucleus of H3K27me3 foci of CTRL and HGPS fibroblasts. Horizontal lines within the boxes represent the medians, upper and lower bounds of the boxes represent quartiles Q3 (75th percentile) and Q1 (25th percentile), respectively, and the whiskers min to max. Number of analyzed nuclei over 3 independent biological replicates: CTRL001-P22: 221; CTRL002-P17: 339; CTRL004-P19: 165. Number of analyzed nuclei over 2 independent biological replicates: HGPS164-P11: 251; HGPS169-P11: 129. Comparisons were tested using two-tail t-test in (b, d). We applied one-way nested ANOVA (test on F statistic) and generalised linear model with Poisson distribution (two-tail test on beta coefficient) in (f, h) and they were both not significant. n.s.: not significant.

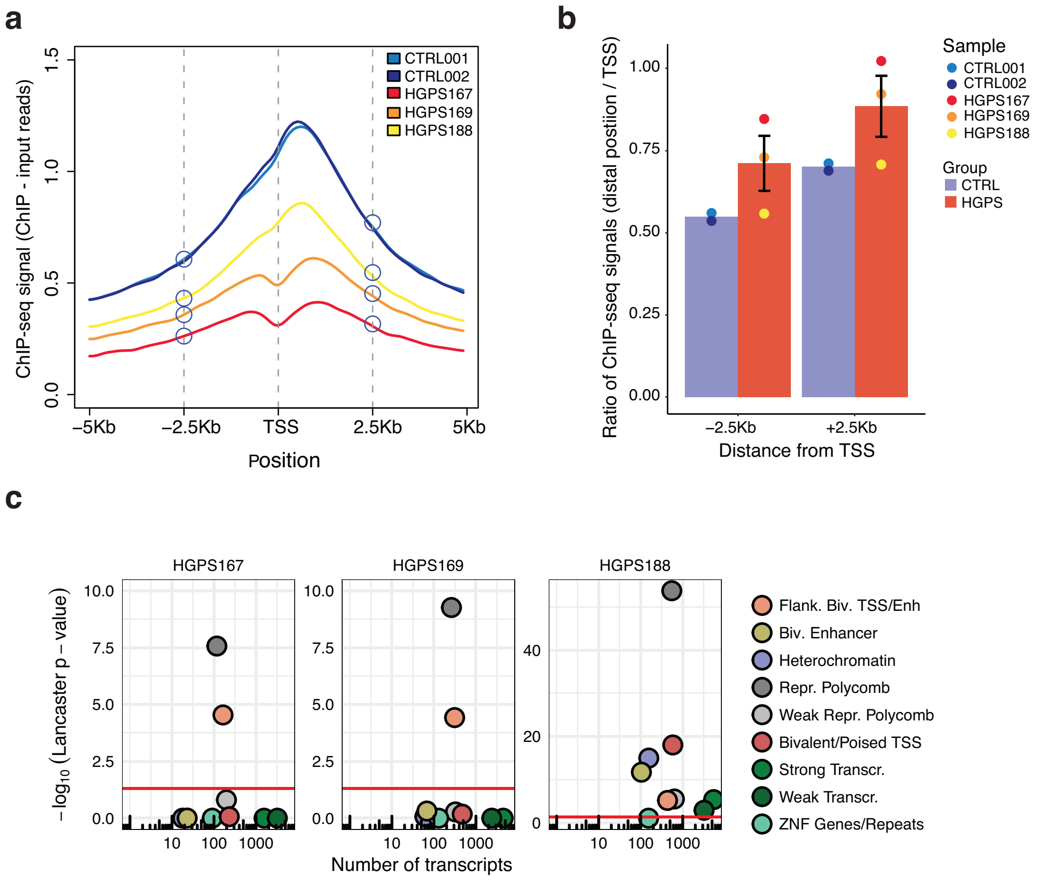

Supplementary Figure 9 - Additional analysis on H3K27me3 patterns. a, H3K27me3 ChIP-seq signal distribution around the TSS region of protein coding genes with H3K27me3 enrichment (4101 genes), based on GENCODE v27 annotation, calculated by deepTools using the genome-wide signal from SPP (see Online methods for details). The x-axis represents relative genomic position around the TSS (+/- 5Kb), and the y-axis represents average signal intensity. **b** For each metaprofile in panel "**a**" we computed the ratio of average ChIP-seq signal at +/-2.5Kb from TSS over the average signal at TSS. The barplots show the average of these ratios for control and HGPS samples. The whiskers mark +/- Standard error. The ratio for each biologically independent sample is marked with a dot of different color. **c**, Transcripts differential expression (up-regulation) was assessed by comparing individual HGPS samples against the group of controls. A two-sided Wald test was used to compute p-values for the differential expression at transcript level, then uncorrected p-values were aggregated based on their chromatin state using the Lancaster method, considering only genes in SAMMY-seq domains (S4 vs S3) (see methods for details). The plot reports the number of transcripts (x-axis) and significance (Lancaster method aggregated p-values – y-axis) for each chromatin state (color legend) as defined by Roadmap Epigenomics[14]. The horizontal red lines mark the 0.05 p-value threshold.

Supplementary Methods

The algorithm performs the segmentation of cell nuclei and the detection of PcG bodies of each image. It then measures the area and eccentricity of nuclei as well as the number and the area of the PcG bodies. It also provides for each PcG body a measure of its closeness to nuclear periphery (proximity). The algorithm has been implemented in MATLAB following this pseudocode (https://github.com/talisman4/2D-PcG-bodies-cell-image-analysis-HGP-Syndrome):

```
[nuclei,avg_Bmi1] = nuclei_seg(I_dapi,lamin, I_Bmi1) %Performs nuclei segmentation
I_avg = imfilter(I_Bmi1,fspecial('average', [7,7]))
I_filt = I_Bmi1 - I_avg
[pcg,t] = isodata_thresh(I_filt,avg_Bmi1) %Applies ISODATA method to separate PcG bodies from nuclei regions
nuclei_CC = bwconncomp(nuclei)
nuclei_L = labelmatrix(nuclei_CC)
compute area for each nucleus object in nuclei_CC
exclude nuclei whose area is less than 10% of mean areas
{NCL}_M <- identified nuclei
for each nucleus m in {NCL}_M
     NCL_m.metric = 4*pi*NCL_m.area/NCL_m.perimeter^2 %compute eccentricity of nucleus NCL_m to provide an estimate of its 'roundness'
     NCL_m.PcG = pcg .* {NCL}_M %positions of detected PcG bodies within the nucleus NCL_m
     NCL_m.PcG = bwareaopen(NCL_m.PcG,3,8)
     PcG_CC = bwconncomp(NCL_m.PcG)
     compute number N of PcG bodies in PcG_CC
     for each PcG body n in NCL_m.{PcG_CC}_N
          NCL_m.PcG_n.area = area of PcG body
          NCL_m.PcG_n.mindist = minimum distance of PcG body from the nuclear periphery
          NCL_m.PcG_n.closestP = the point on nuclear periphery closest to PcG body
          NCL_m.PcG_n.distC2P = distance of nuclear centroid from closestP
          NCL_m.PcG_n.proximity = NCL_m.PcG_n.mindist / NCL_m.PcG_n.distC2P
     endfor
endfor
```

$I_{dapi,lamin}$ is the image obtained by the sum of both images showing the fluorescence of nucleus and lamin.

The function *nuclei_seg* performs a partition of cell image $I_{dapi,lamin}$ in nuclei regions and background implementing a region based segmentation algorithm[15]. `nuclei` is the binary image defining nuclei regions while avg_{Bmi1} is the mean intensity value of the nuclei regions in the image I_{Bmi1} that shows the fluorescence of PcG bodies.

In order to better enhance PcG areas we subtract from the original image I_{Bmi1} its smoothed version obtained by applying an averaging filter of size 7, producing the image I_{filt}.

The function *isodata_thresh* implements the ISODATA classification algorithm[16] and uses relevant values computed by *nuclei_seg* function in order to extract PcG bodies from the

nuclei regions. It sets the initial threshold value of ISODATA method as avg_{Bmi1}. pcg is the binary image defining PcG bodies.
Reconstructions of nuclei are obtained through the connected components algorithm (*bwconncomp* MATLAB function, using a connectivity of 8). Nuclei are then labeled by applying the *labelmatrix* MATLAB function so they can be easily separated from each other.
The algorithm computes the area of each nucleus, discarding objects whose area is less than 10% of mean areas which are just noise.
The algorithm uses the *bwareaopen* function in order to discard too small detected PcG objects which are probably just noise.
Reconstructions of PcG bodies are obtained through the connected components algorithm (*bwconncomp* MATLAB function, using a connectivity of 6).

Supplementary references

1. Henikoff, S., Henikoff, J.G., Sakai, A., Loeb, G.B. & Ahmad, K. Genome-wide profiling of salt fractions maps physical properties of chromatin. *Genome Res* **19**, 460-9 (2009).
2. Baudement, M.O. et al. High-salt-recovered sequences are associated with the active chromosomal compartment and with large ribonucleoprotein complexes including nuclear bodies. *Genome Res* **28**, 1733-1746 (2018).
3. Dou, Z. et al. Autophagy mediates degradation of nuclear lamina. *Nature* **527**, 105-9 (2015).
4. Lund, E., Oldenburg, A.R. & Collas, P. Enriched domain detector: a program for detection of wide genomic enrichment domains robust against local variations. *Nucleic Acids Res* **42**, e92 (2014).
5. Lund, E.G., Duband-Goulet, I., Oldenburg, A., Buendia, B. & Collas, P. Distinct features of lamin A-interacting chromatin domains mapped by ChIP-sequencing from sonicated or micrococcal nuclease-digested chromatin. *Nucleus* **6**, 30-9 (2015).
6. McCord, R.P. et al. Correlated alterations in genome organization, histone methylation, and DNA-lamin A/C interactions in Hutchinson-Gilford progeria syndrome. *Genome Res* **23**, 260-9 (2013).
7. Sadaie, M. et al. Redistribution of the Lamin B1 genomic binding profile affects rearrangement of heterochromatic domains and SAHF formation during senescence. *Genes Dev* **27**, 1800-8 (2013).
8. van Steensel, B. & Henikoff, S. Identification of in vivo DNA targets of chromatin proteins using tethered dam methyltransferase. *Nat Biotechnol* **18**, 424-8 (2000).
9. Becker, J.S. et al. Genomic and Proteomic Resolution of Heterochromatin and Its Restriction of Alternate Fate Genes. *Mol Cell* **68**, 1023-1037 e15 (2017).
10. Nicetto, D. et al. H3K9me3-heterochromatin loss at protein-coding genes enables developmental lineage specification. *Science* **363**, 294-297 (2019).
11. Spracklin, G. & Pradhan, S. Protect-seq: genome-wide profiling of nuclease inaccessible domains reveals physical properties of chromatin. *Nucleic Acids Res* **48**, e16 (2020).
12. Schmidl, C., Rendeiro, A.F., Sheffield, N.C. & Bock, C. ChIPmentation: fast, robust, low-input ChIP-seq for histones and transcription factors. *Nat Methods* **12**, 963-965 (2015).
13. O'Neill, L.P. & Turner, B.M. Immunoprecipitation of native chromatin: NChIP. *Methods* **31**, 76-82 (2003).
14. Roadmap Epigenomics, C. et al. Integrative analysis of 111 reference human epigenomes. *Nature* **518**, 317-30 (2015).
15. Goldstein, T., Bresson, X. & Osher, S. Geometric Applications of the Split Bregman Method: Segmentation and Surface Reconstruction. *Journal of Scientific Computing* **45**, 272-293 (2010).
16. Ball, G. & Hall, D. ISODATA: A novel method of data analysis and pattern classification. (Stanford Research Institute, Menlo Park, 1965).
