# Supplementary material for: SAMMY-seq reveals early alteration of heterochromatin and deregulation of bivalent genes in Hutchinson-Gilford Progeria Syndrome
Source: Nat Commun. 2020 Dec 8;11:6274. doi: 10.1038/s41467-020-20048-9 (PMC7722762; doi:10.1038/s41467-020-20048-9)
Supplement: Supplementary file 3 — Reporting summary [file 41467_2020_20048_MOESM3_ESM.pdf]

nature research

Corresponding author(s): Chiara Lanzuolo

Last updated by author(s): Sep 19, 2020

Reporting Summary

Nature Research wishes to improve the reproducibility of the work that we publish. This form provides structure for consistency and transparency in reporting. For further information on Nature Research policies, see our Editorial Policies and the Editorial Policy Checklist.

Statistics

For all statistical analyses, confirm that the following items are present in the figure legend, table legend, main text, or Methods section.

n/a	Confirmed	
☐	☒	The exact sample size (*n*) for each experimental group/condition, given as a discrete number and unit of measurement
☐	☒	A statement on whether measurements were taken from distinct samples or whether the same sample was measured repeatedly
☐	☒	The statistical test(s) used AND whether they are one- or two-sided<br>*Only common tests should be described solely by name; describe more complex techniques in the Methods section.*
☐	☒	A description of all covariates tested
☐	☒	A description of any assumptions or corrections, such as tests of normality and adjustment for multiple comparisons
☐	☒	A full description of the statistical parameters including central tendency (e.g. means) or other basic estimates (e.g. regression coefficient) AND variation (e.g. standard deviation) or associated estimates of uncertainty (e.g. confidence intervals)
☐	☒	For null hypothesis testing, the test statistic (e.g. *F*, *t*, *r*) with confidence intervals, effect sizes, degrees of freedom and *P* value noted<br>*Give P values as exact values whenever suitable.*
☒	☐	For Bayesian analysis, information on the choice of priors and Markov chain Monte Carlo settings
☒	☐	For hierarchical and complex designs, identification of the appropriate level for tests and full reporting of outcomes
☐	☒	Estimates of effect sizes (e.g. Cohen's *d*, Pearson's *r*), indicating how they were calculated

Our web collection on statistics for biologists contains articles on many of the points above.

Software and code

Policy information about availability of computer code

Data collection	NIS Element Digital Software and LAS-AF software (version 2.6.0) was used for image acquisition. 2100 Bioanalyzer Expert Software (version 2100 Expert Software B.02.08 SI648) package was used to analyse library quality before sequencing.
Data analysis	Cell Profiler 2.0 was used for PLA blobs quantification, Matlab 8.4 and Matlab custom code for detecting and quantifying PcG bodies in fluorescence cell images. For sequencing data analyses: FASTQC 0.11.5, bwa 0.7.12 and 0.7.17 (the updated subversion was used in specific analyses as detailed in the text), Trimmomatic 0.32, samtools 1.3.1 and 1.9 (scale-down and late passages analysis), biobambam2 2.0.54, bedtools 2.25.0 and 2.29.0 (the updated subversion was used in specific analyses as detailed in the text), bedgraphToBigwig, wigToBigWig, bigWigToBedGrap and liftOver version 4, SPP 1.15.4 and 1.16.0 (scale-down analysis), EDD (version 1.1.15 and 1.1.19) (the updated subversion was used in specific analyses as detailed in the text), R 3.3.1 and 3.5.1 (the updated subversion was used in specific analyses as detailed in the text). mgcv 1.8-12, Gviz (version 1.26.5), karyoploter (version 1.2.2), ggplot2 (version 3.3.2), edgeR (version 3.24.3) R packages. deepTools 3.2.1 and 3.3.2 (the updated subversion was used in specific analyses as detailed in the text), StereoGene 1.73 and 2.20 (the updated subversion was used in specific analyses as detailed in the text), Kallisto 0.43.0, sleuth R package (0.29.0), aggregate R package (1.0.1)

For manuscripts utilizing custom algorithms or software that are central to the research but not yet described in published literature, software must be made available to editors and reviewers. We strongly encourage code deposition in a community repository (e.g. GitHub). See the Nature Research guidelines for submitting code & software for further information.

Data

Policy information about availability of data

All manuscripts must include a data availability statement. This statement should provide the following information, where applicable:

- Accession codes, unique identifiers, or web links for publicly available datasets
- A list of figures that have associated raw data
- A description of any restrictions on data availability

The high-throughput sequencing data generated for this study are available in the GEO repository with accession number GSE118633 [https://www.ncbi.nlm.nih.gov/geo/query/acc.cgi?acc=GSE118633]. Other previously published genomics data used in this article were released in public repositories by the original publication authors as indicated in the article or Methods details above. These include GEO datasets for ATAC-seq (GSE80639 [https://www.ncbi.nlm.nih.gov/geo/query/acc.cgi?acc=GSE80639]), Lamin A/C ChIP-seq (GSE41757 [https://www.ncbi.nlm.nih.gov/geo/query/acc.cgi?acc=GSE41757] and GSE54332 [https://www.ncbi.nlm.nih.gov/geo/query/acc.cgi?acc=GSE54332]), Lamin B1 ChIP-seq (GSE49341 [https://www.ncbi.nlm.nih.gov/geo/query/acc.cgi?acc=GSE49341] and GSE63440 [https://www.ncbi.nlm.nih.gov/geo/query/acc.cgi?acc=GSE63440]), in addition to Roadmap Epigenomics consolidated ChIP-seq datasets for E055 (human foreskin fibroblasts) for histone marks (H3K9me3, H3K27me3, H3K4me1, H3K36me3, H3K27ac and H3K4me3) retrieved from the Roadmap Epigenomics on line repository at URL [https://egg2.wustl.edu/roadmap/data/byFileType/alignments/consolidated].
The source data underlying Figures 2b, 2c, 3b, 3c, 4b, 4c, 4d, 5a, 5c, 5d, 6a, 6d, 6e, 6f and Supplementary Figures 1b, 1c, 1d, 1e, 1f, 1g, 1h, 1i, 2a, 2b, 2c, 3b, 3c, 4c, 4e, 4f, 5b, 5c, 5d, 5f, 5g, 5h, 6a, 6b, 6d, 6e, 7a, 7b, 7c, 7d, 8b, 8d, 8f, 8h, 9a, 9b, 9c are provided as a Source Data file.

Field-specific reporting

Please select the one below that is the best fit for your research. If you are not sure, read the appropriate sections before making your selection.

- [x] Life sciences
- [ ] Behavioural & social sciences
- [ ] Ecological, evolutionary & environmental sciences

For a reference copy of the document with all sections, see nature.com/documents/nr-reporting-summary-flat.pdf

Life sciences study design

All studies must disclose on these points even when the disclosure is negative.

Sample size	We didn't perform power calculation as only a limited number of early passage primary fibroblasts were available from the Progeria Research Foundation biobank. For this reason, we used up to 3 independent biological replicates for Hutchinson Gilford Progeria Syndrome patients derived primary fibroblasts. We used the same number of healthy control primary fibroblasts to match the progeria samples number.
Data exclusions	ChIP-seq for H3K27me3 was performed on three independent control samples for primary fibroblasts, but one of them failed.
Replication	For all experiments 2 or 3 replicates were used (as indicated in individual figures and results). All attempts at replication were successful, unless a ChIP-seq for H3K27me3 as reported above.
Randomization	Not applicable as we are comparing two different genotypes (wild type and progeria), thus there is no randomization of tratment.
Blinding	Not applicable as we are comparing two different genotypes (wild type and progeria), thus there is no blinding of treatment.

Reporting for specific materials, systems and methods

We require information from authors about some types of materials, experimental systems and methods used in many studies. Here, indicate whether each material, system or method listed is relevant to your study. If you are not sure if a list item applies to your research, read the appropriate section before selecting a response.

Materials & experimental systems

n/a	Involved in the study	
	x	Antibodies
	x	Eukaryotic cell lines
x		Palaeontology and archaeology
x		Animals and other organisms
x		Human research participants
x		Clinical data
x		Dual use research of concern

Methods

n/a	Involved in the study	
	x	ChIP-seq
x		Flow cytometry
x		MRI-based neuroimaging

Antibodies

Antibodies used	ChIP-seq: H3K9me3 antibody (ab8898, Abcam) and H3K27me3 (07-449, Millipore).

Western blot: Anti-tubulin alpha (Sigma T5168, mouse 1:10000), H3 (Abcam ab1791, rabbit 1:4000), Beta-Actin (Santa-Cruz sc1616, rabbit 1:4000), Lamin A/C (Santa Cruz sc-6215, goat 1:4000), Lamin B (Santa Cruz sc-6216, goat 1:2000), progerin (13A4 mouse, Abcam 66587, mouse 1:1000), Ezh2 (AC22 Cell Signaling 3147S, mouse 1:1000), Bmi1 (D42B3 Cell signaling, rabbit 1:1000), H3K9me3 (Abcam ab8898, rabbit 1:1000), H3K27me3 (Millipore 07-449, rabbit 1:1000).
Anti-Mouse IgG-Peroxidase (Sigma, A9044), 1:1000 in 5% milk
Anti-Rabbit IgG-Peroxidase (Sigma, A9169), 1:2000 in 5% milk
Anti-Goat IgG-Peroxidase (Sigma, A5420), 1:5000 in 5% milk
Immunofluorescence: Bmi1 (Millipore 05-637, mouse) diluted 1:100; Lamin A/C (Santa Cruz sc-6215, goat) diluted 1:200; Ezh2 (Cell signaling AC22 3147S, mouse) diluted 1:100; H3K9me3 (Abcam ab8898, rabbit) diluted 1:500; H3K27me3 (Millipore 07-449, rabbit) diluted 1:100.
Alexa Fluor 488 Donkey anti-rabbit IgG (Jackson ImmunoResearch Laboratories, 711-545-152)
Alexa Fluor 647 Chicken anti-goat IgG (Invitrogen, A21469)
Alexa Fluor 594 Donkey anti-goat IgG (Jackson ImmunoResearch Laboratories, 705-585-003)
Alexa Fluor 488 Donkey anti-mouse IgG (Jackson ImmunoResearch Laboratories, 715-545-150)
Proximity Ligation Assay (PLA): progerin (Alexis human mAb, 13A4, ALX-804-662-R200) diluted 1:20; Lamin A/C (Santa Cruz sc-6215, goat) diluted 1:200; Ezh2 (Cell signaling 4905S, rabbit 1:100); Bmi1 (Abcam ab85688 rabbit 1:100).
BrdU antibody (1:10, Becton Dickinson 347580)

Validation

All primary antibodies used in this study were validated by manufacturers and validation statement for each antibody is provided on the manufacture's website.

H3K9me3 (Abcam ab8898, rabbit). Abcam website antibody validation and more than 1000 references. Used for ChIP-seq in Encode project. IF validation on Hela cells according to Abcam IF protocol. Western Blot validation on Mouse Tissue lysate - whole (Heart), detecting 1 band of 17 kDa.
Citation from manufacturer are listed at:
https://www.abcam.com/histone-h3-tri-methyl-k9-antibody-chip-grade-ab8898.html

H3K27me3 (Millipore 07-449, rabbit). Sigma Aldrich website antibody validation and several references. The purified antibody is dot blot tested for trimethylated lysine 27 specificity. Used for ChIP-seq in Encode project. IF validation on Mouse embryonic fibroblasts. Western Blot validation on Hela cells, detecting 1 band of 17 kDa.
Citation from manufacturer are listed at:
https://www.merckmillipore.com/IT/it/product/Anti-trimethyl-Histone-H3-Lys27-Antibody,MM_NF-07-449

Anti-tubulin alpha (Sigma T5168, mouse). Sigma Aldrich website antibody validation and several references. Western Blot validation on Hela, Jurkat, COS7, NIH-3T3, PC12, RAT2, CHO, MDBK and MDCK cells, detecting 1 band of 50 kDa.
Citation from manufacturer are listed at:
https://www.sigmaaldrich.com/catalog/product/sigma/t5168?lang=it&region=IT&gclid=CjwKCAjw2Jb7BRBHEiwAXTR4jcHyxWaLCH5h1_TcDBPTOzoRJZ0X3k4bRIaPvjLJGfDh7pOpOIu_DxoC3dsQAvD_BwE

H3 (Abcam ab1791, rabbit 1:4000). Abcam website antibody validation and more than 1000 references. Western Blot validation on Mouse Tissue lysate - whole (Heart), detecting 1 band of 17 kDa.
Citation from manufacturer are listed at:
https://www.abcam.com/histone-h3-tri-methyl-k9-antibody-chip-grade-ab8898.html

Beta-Actin (Santa-Cruz sc1616). Santa Cruz website antibody validation and more than 1000 references. Western Blot validation on C32, BC3H1, Sol 8 and L8 whole cell lysates, detecting 1 band of 43 kDa.
Citation from manufacturer are listed at:
https://www.scbt.com/it/p/actin-antibody-i-19

Lamin A/C (Santa Cruz sc-6215). Santa Cruz website antibody validation and more than 100 references. Western Blot validation on 293T, Lamin A transfected 293T and Hs58 cells, detecting 1 band of 62/69 kDa. Immunofluorescence validation on transgenic Drosophila salivary gland nucleus expressing human Lamin A. The product has been discontinued.
Citation from manufacturer are listed at:
https://www.scbt.com/it/p/lamin-a-c-antibody-n-18

Lamin B (Santa Cruz sc-6216, goat 1:2000). Santa Cruz website antibody validation and more than 200 references. Western Blot validation on CCRF-CEM, detecting 1 band of 67 kDa. Immunofluorescence validation on methanol-fixed F9 cells. The product has been discontinued.
Citation from manufacturer are listed at:
https://www.scbt.com/it/p/lamin-b-antibody-c-20

progerin/western blot:
progerin (13A4 mouse, Abcam 66587). Abcam website antibody validation and 10 references. Western Blot validation on Hela cells ectopically expressing Flag-progerin, detecting 1 band of 70 kDa.
Citation from manufacturer are listed at:
https://www.abcam.com/progerin-antibody-13a4-ab66587.html#description_images_1

progerin/ Proximity Ligation Assay:
progerin (Alexis human mAb, 13A4, ALX-804-662-R200). Labome website antibody validation and 6 references. Validated by the

manufacturer for immunocytochemistry.
Citation from manufacturer are listed at:
https://www.labome.com/product/Enzo-Life-Sciences/ALX-804-662-R200.html

Ezh2/western blot/immunofluorescence:
Ezh2 (AC22 Cell Signaling 3147S). Cell Signaling website antibody validation and 100 references. Western Blot validation on T47D, MCF7, SEM and MDA-MB-134 cells, detecting 1 band of 98 kDa. Immunofluorescence validation on Hela cells.
Citation from manufacturer are listed at:
https://www.cellsignal.com/products/primary-antibodies/ezh2-ac22-mouse-mab/3147?Ntk=Products&Ntt=3147

Ezh2/ Proximity Ligation Assay:
Ezh2 (Cell signaling 4905S). Cell Signaling website antibody validation and 59 references.
Citation from manufacturer are listed at:
https://www.cellsignal.com/products/primary-antibodies/ezh2-antibody/4905?Ntk=Products&Ntt=4905

Bmi1/western blot:
Bmi1 (D42B3 Cell signaling). Cell Signaling website antibody validation and 21 references. Western Blot validation on Hela, NIH-3T3, H-4-II-E, COS-7 cells, detecting 1 band of 41 kDa.
Citation from manufacturer are listed at:
https://www.cellsignal.com/products/primary-antibodies/bmi1-d42b3-rabbit-mab/5856?Ntk=Products&Ntt=5856

Bmi1/immunofluorescence:
Bmi1 (Millipore 05-637). Merk Millipore website antibody validation and several references. Validated by the manufacturer for immunocytochemistry.
Citation from manufacturer are listed at:
https://www.merckmillipore.com/IT/it/product/Anti-Bmi-1-Antibody-clone-F6,MM_NF-05-637

Bmi1/Proximity Ligation Assay:
Bmi1 (Abcam ab85688). Abcam website antibody validation and 5 references. Immunohistochemistry validation on human lung carcinoma tissue.
Citation from manufacturer are listed at:
https://www.abcam.com/bmi1-antibody-ab85688.html

Anti-Mouse IgG-Peroxidase (Sigma, A9044), 1:1000 in 5% milk
Anti-Mouse IgG (whole molecule)–Peroxidase antibody produced in rabbit (Sigma, Cat # A9044). Citation from manufacturer are listed at https://www.sigmaaldrich.com/catalog/product/sigma/a9044

Anti-Rabbit IgG-Peroxidase (Sigma, A9169), 1:2000 in 5% milk
Anti-Rabbit IgG (whole molecule)–Peroxidase antibody produced in goat (Sigma, Cat # A9169). Citation from manufacturer are listed at https://www.sigmaaldrich.com/catalog/product/sigma/a9169

Anti-Goat IgG-Peroxidase (Sigma, A5420), 1:5000 in 5% milk
Anti-Goat IgG (whole molecule)–Peroxidase antibody produced in rabbit (Sigma, Cat# A5420). Citation from manufacturer are listed at https://www.sigmaaldrich.com/catalog/product/sigma/a5420

Alexa Fluor 488 Donkey anti-rabbit IgG (Jackson ImmunoResearch Laboratories, 711-545-152)
Alexa Fluor® 488 AffiniPure Donkey Anti-Rabbit IgG (H+L) (Jackson ImmunoResearch Laboratories, Cat# 711-545-152). Citation from manufacturer are listed at https://www.jacksonimmuno.com/catalog/products/711-545-152.

Alexa Fluor 647 Chicken anti-goat IgG (Invitrogen, A21469)
Chicken anti-Goat IgG (H+L) Cross-Adsorbed Secondary Antibody, Alexa Fluor 647 (Invitrogen, Cat# A21469). Citation from manufacturer are listed at https://www.thermofisher.com/antibody/product/Chicken-anti-Goat-IgG-H-L-Cross-Adsorbed-Secondary-Antibody-Polyclonal/A-21469.

Alexa Fluor 594 Donkey anti-goat IgG (Jackson ImmunoResearch Laboratories, 705-585-003)
Alexa Fluor® 594 AffiniPure Donkey Anti-Goat IgG (H+L) (Jackson ImmunoResearch Laboratories, Cat# 705-585-003). Citation from manufacturer are listed at https://www.jacksonimmuno.com/catalog/products/705-585-003

Alexa Fluor 488 Donkey anti-mouse IgG (Jackson ImmunoResearch Laboratories, 715-545-150)
Alexa Fluor® 488 AffiniPure Donkey Anti-Mouse IgG (H+L) (Jackson ImmunoResearch Laboratories, Cat#715-545-150). Citation from manufacturer are listed at https://www.jacksonimmuno.com/catalog/products/715-545-150.

BrdU antibody (Becton Dickinson 347580). Bioscience website antibody validation and several references. Validated by the manufacturer for immunocytochemistry.
Citation from manufacturer are listed at:
https://www.bdbiosciences.com/us/applications/research/apoptosis/purified-antibodies/purified-mouse-anti-brdu-b44/p/347580

Eukaryotic cell lines

Policy information about cell lines

Cell line source(s)	HGADFN164 (HGPS164), HGADFN167 (HGPS167), HGADFN169 (HGPS169), HGADFN188 (HGPS188), HGADFN271 (HGPS271) human dermal fibroblasts derived from HGPS patients were provided by the Progeria Research Foundation (PRF). AG08498 (CTRL001) and AG07095 (CTRL002) human dermal fibroblasts were obtained from the Coriell Institute. Foreskin fibroblast strain #2294 (CTRL004) was a generous gift from the Laboratory of Molecular and Cell Biology, Istituto Dermopatico dell'Immacolata (IDI)-IRCCS, Rome, Italy", while control dermal fibroblast CTRL013 was kindly provided by the Italian Laminopathies Network.
Authentication	Control and progeric fibroblasts used in this study were tested for the presence of the single nucleotide mutation by PCR of the DNA fragment spanning the mutation site followed by sequence.
Mycoplasma contamination	We tested all cell lines for mycoplasma contamination by PCR each 6 months, using the following primers: 5'-ACT CCT ACG GGA GGC AGC AGT A-3'; 5'-TGC ACC ATC TGT CAC TCT GTT AAC CTC-3'. A positive control was used in the PCR amplification.
Commonly misidentified lines (See ICLAC register)	We did not use misidentified cell lines

ChIP-seq

Data deposition

☒ Confirm that both raw and final processed data have been deposited in a public database such as GEO.

☒ Confirm that you have deposited or provided access to graph files (e.g. BED files) for the called peaks.

Data access links *May remain private before publication.*	GEO dataset GSE118633; reviewers can access anonymously the data using this token: cjstcccyhjsdnox at URL: https://www.ncbi.nlm.nih.gov/geo/query/acc.cgi?acc=GSE118633
Files in database submission	Raw sequencing files, Bigwig files for genomics tracks and BED files for peak calls
Genome browser session (e.g. UCSC)	N/A

Methodology

Replicates	H3K9me3 ChIP-seq were done on 3 distinct control- and 3 progeric patient-derived fibroblasts. H3K27me3 ChIP-seq were done on 2 distinct control- and 3 progeric-derived fibroblasts.

Sequencing depth

All H3K9me3 reads were 50bp single end and all H3K27me3 reads were 50bp paired end, where we only used the R1 read.
total reads
H3K9me3 IP
CTRL002 CTRL004 CTRL013 HGPS167 HGPS169 HGPS188
34058960 46032537 33447825 34919775 51888722 36678922

H3K9me3 input
CTRL002 CTRL004 CTRL013 HGPS167 HGPS169 HGPS188
38503431 64635536 51043909 36220262 40283830 38274998

H3K27me3 IP
CTRL001 CTRL002 HGPS167 HGPS169 HGPS188
33282519 40211798 31549201 33459533 34609911

H3K27me3 input
CTRL001 CTRL002 HGPS167 HGPS169 HGPS188
32037829 34616588 23891447 34823229 35793218
uniquely mapped reads (also excluding X and Y chromosomes)

H3K9me3 IP
CTRL002 CTRL004 CTRL013 HGPS167 HGPS169 HGPS188
22395757 28347416 20770495 22670188 30923759 23049546

H3K9me3 input
CTRL002 CTRL004 CTRL013 HGPS167 HGPS169 HGPS188
24683985 43105626 32533625 24798209 26984898 25431815

H3K27me3 IP
CTRL001 CTRL002 HGPS167 HGPS169 HGPS188

29194930 35304331 27527560 29446994 30590671

H3K27me3 input
CTRL001 CTRL002 HGPS167 HGPS169 HGPS188
27262533 29641073 15451134 21825680 31088959

Antibodies

H3K9me3 antibody (ab8898, Abcam) and H3K27me3 (07-449, Millipore)

Peak calling parameters

H3K9me3 peak calling with the EDD tool:

```
edd -p 4 --fdr 0.1 --gap-penalty 10 --bin-size 100 --write-log-ratios --write-bin-scores genome_size_file.txt blacklisted_regions.bed chip.bam input.bam > output_dir
```

H3K27me3 peak calling with SPP in R:

```
cchrs <- paste0("chr", c(1:22))
ip_cc <- get.binding.characteristics(ip)
ip_informative    <- select.informative.tags(ip, ip_cc)
input_informative <- select.informative.tags(input, ip_cc)
ip_informative    <- remove.local.tag.anomalies(ip_informative[chrs])
input_informative <- remove.local.tag.anomalies(input_informative[chrs])

broad_regions <- get.broad.enrichment.clusters(ip_informative, input_informative,
                              window.size = 2000, z.thr = 3,
                              tag.shift = round(ip_cc$peak$x / 2))
```

Data quality

We ran FASTQC 0.11.5 on all samples and manually checked for any serious data quality issue. After initial read mapping we discarded all unmapped, PCR duplicate, QCFAIL flagged, MQ = 0 and multimapping reads from further analysis.

The filtered number of peaks we used for downstream analysis is as follows:

H3K9me3 peaks called with EDD

CTRL002 175
CTRL004 173
CTRL013 187
HGPS167 186
HGPS169 175
HGPS188 177

H3K27me3 peaks called with SPP:

CTRL001 53332
CTRL002 55741
HGPS167 159731
HGPS169 145366
HGPS188 68427

Software

FASTQC 0.11.5, bwa 0.7.12, Trimmomatic 0.32, samtools 1.3.1, biobambam2 2.0.54, bedtools 2.25.0, bedgraphToBigwig, wigToBigWig, bigWigToBedGrap and liftOver version 4, SPP 1.15.4, EDD 1.1.15, R 3.3.1 and 3.5.1, mgcv 1.8-12, Gviz, karyoploter, ggplot2 R packages, deepTools 3.2.1, StereoGene 1.73, Kallisto 0.43.0, sleuth R package (0.29.0), aggregate R package (1.0.1)
