# Supplementary material for: SAMMY-seq reveals early alteration of heterochromatin and deregulation of bivalent genes in Hutchinson-Gilford Progeria Syndrome
Source: Nat Commun. 2020 Dec 8;11:6274. doi: 10.1038/s41467-020-20048-9 (PMC7722762; doi:10.1038/s41467-020-20048-9)

FIGURE 6f CTRL004 p17 vs HGPS167 p10

alfa-Tubulin shown in fig.6f

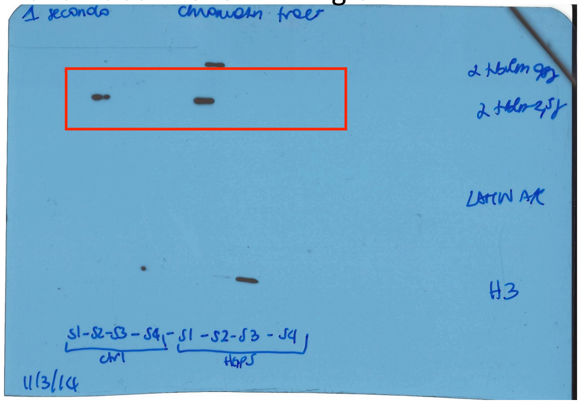

Progerin shown in fig.6f

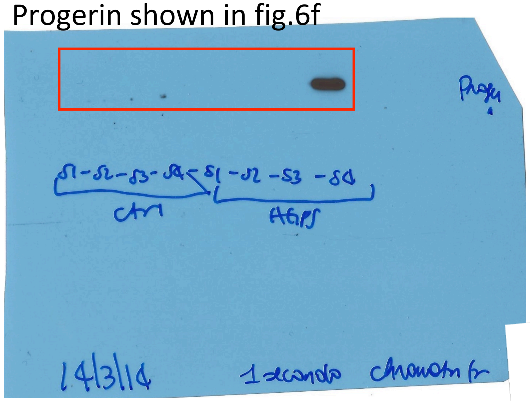

H3 shown in fig.6f

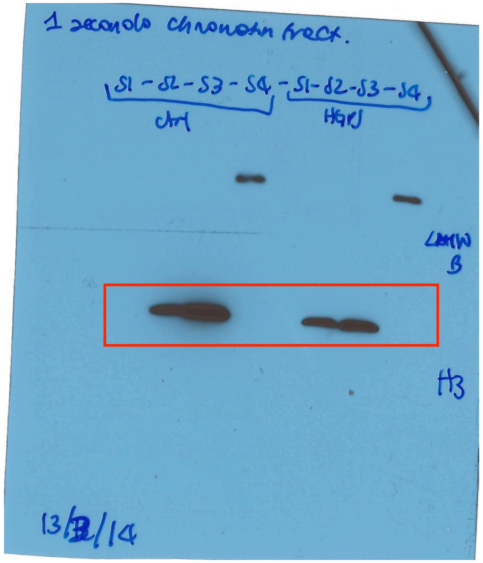

Lamin A/C shown in fig.6f

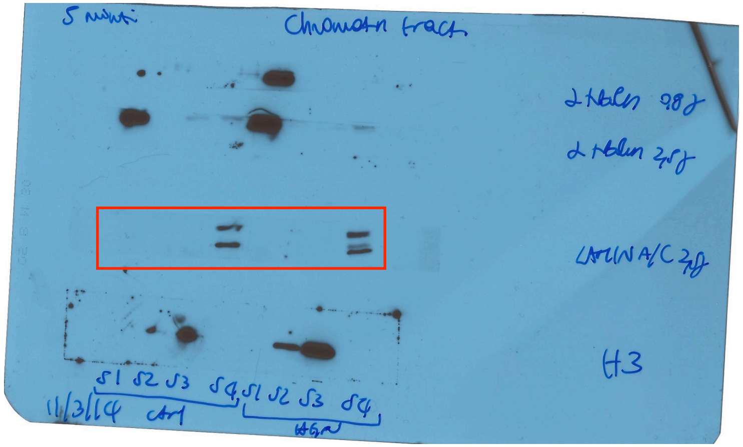

EzH2 shown in fig.6f

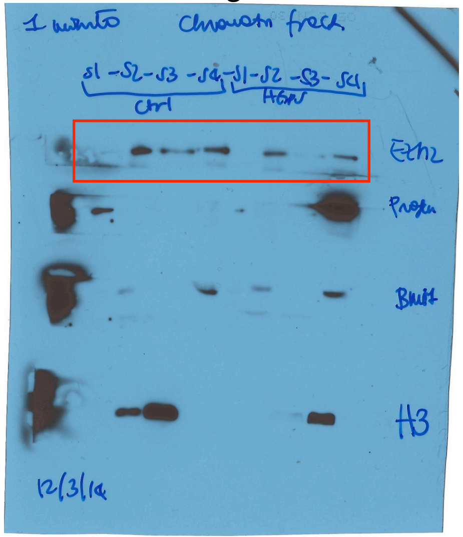

EzH2 shown in fig.6f

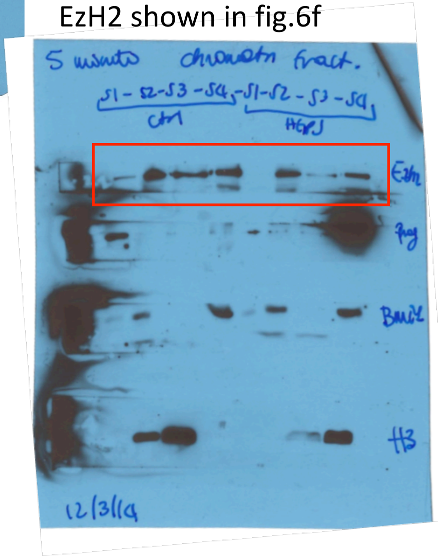

Bmi1 shown in fig.6f

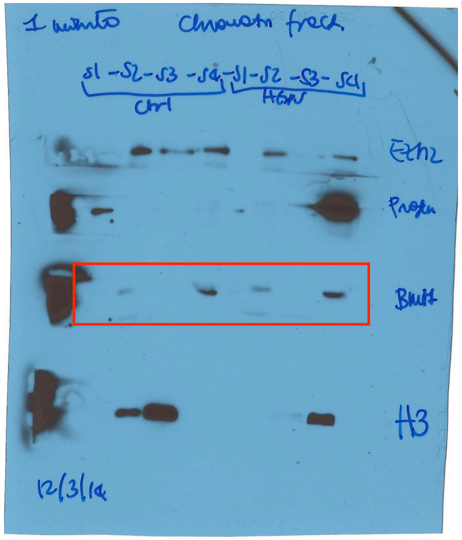

Bmi1 shown in fig.6f

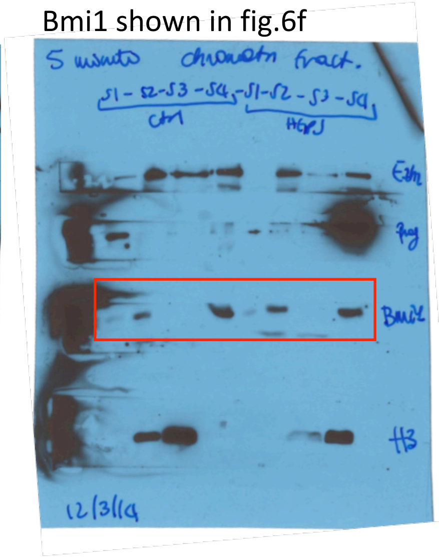

FIGURE 6f CTRL004 p27 vs HGPS167 p20

alfa-Tubulin shown in fig.6f

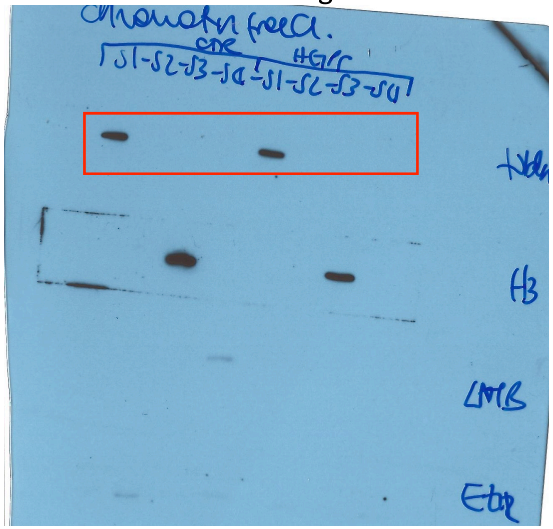

H3 shown in fig.6f

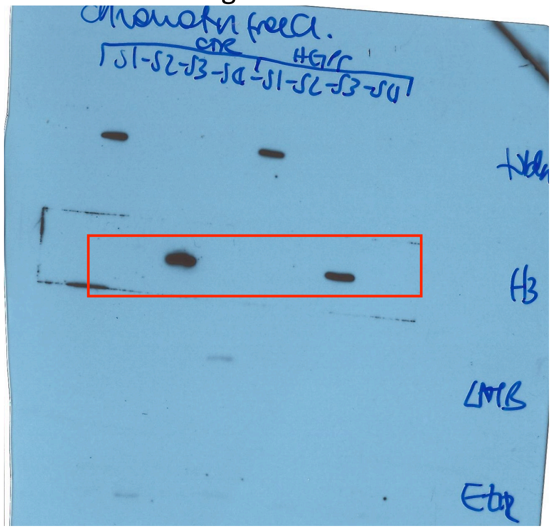

Lamin A/C shown in fig.6f

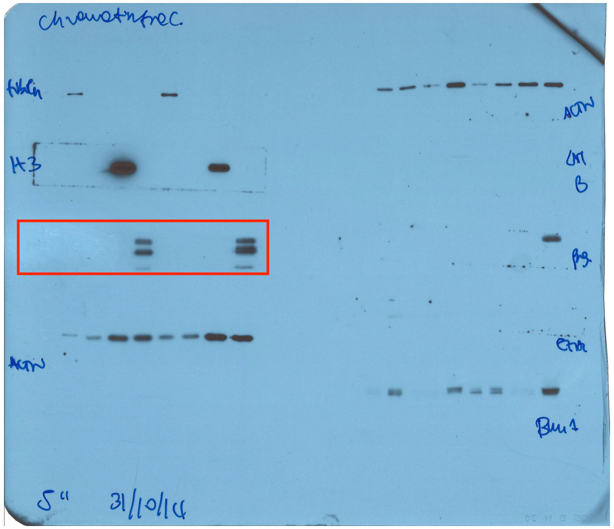

Progerin shown in fig.6f

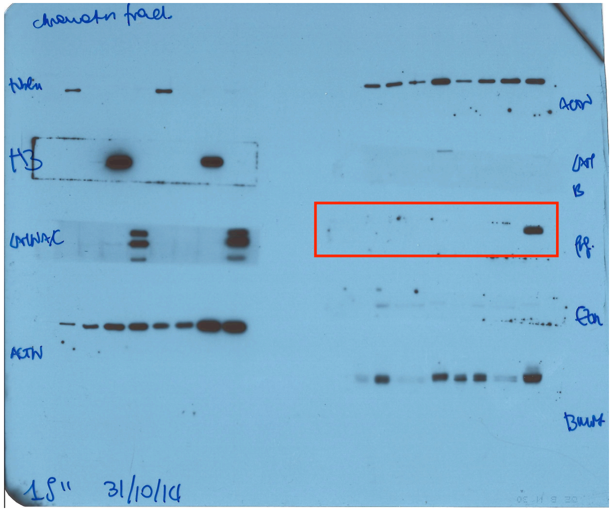

Ezh2 shown in fig.6f

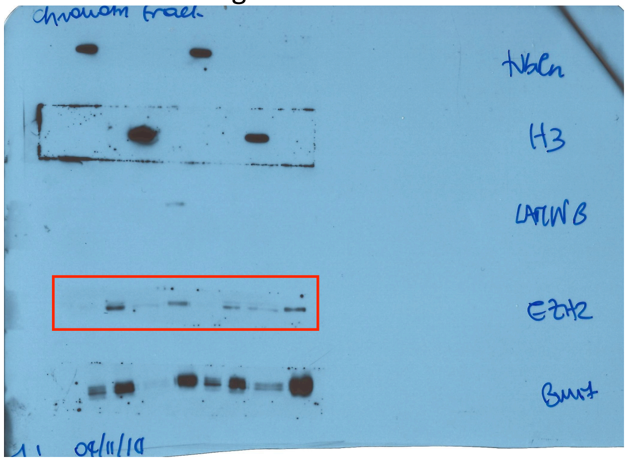

Ezh2 shown in fig.6f

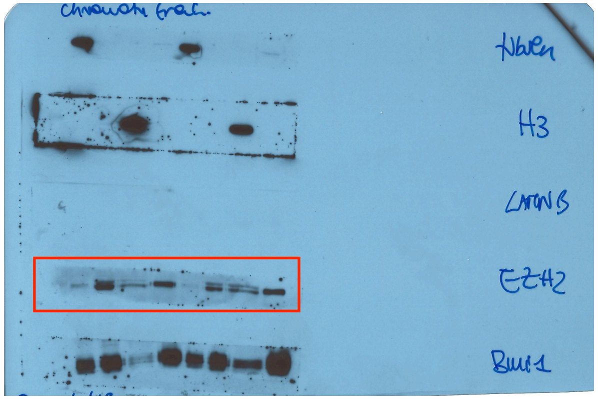

FIGURE 6f CTR004 p27 vs HGPS167 p20

Bmi1 shown in fig.6f

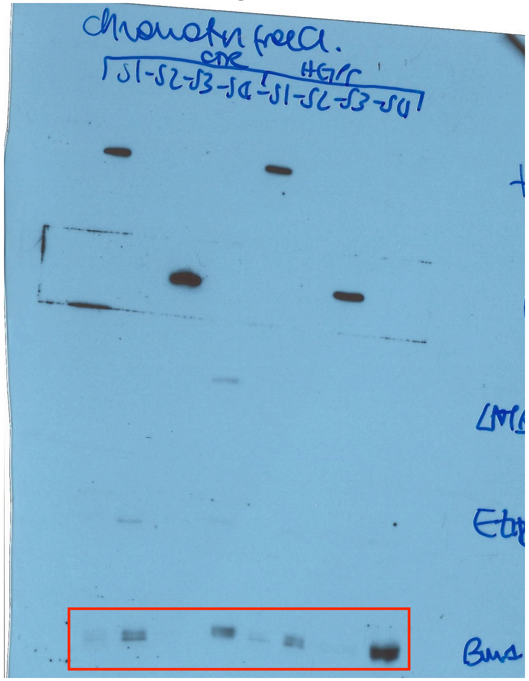

Bmi1 shown in fig.6f

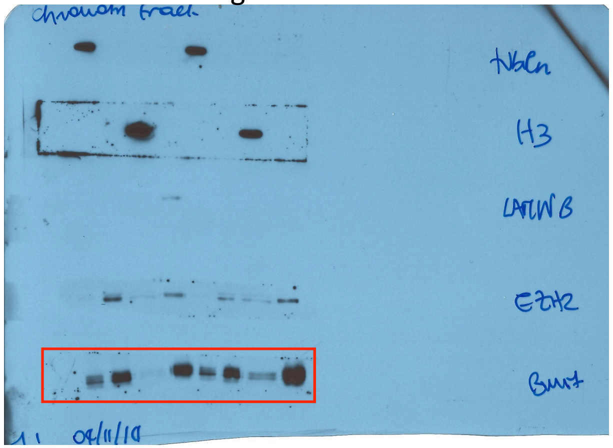

SUPPLEMENTARY FIGURE 1a

Tubulin shown in supplementary fig.1a

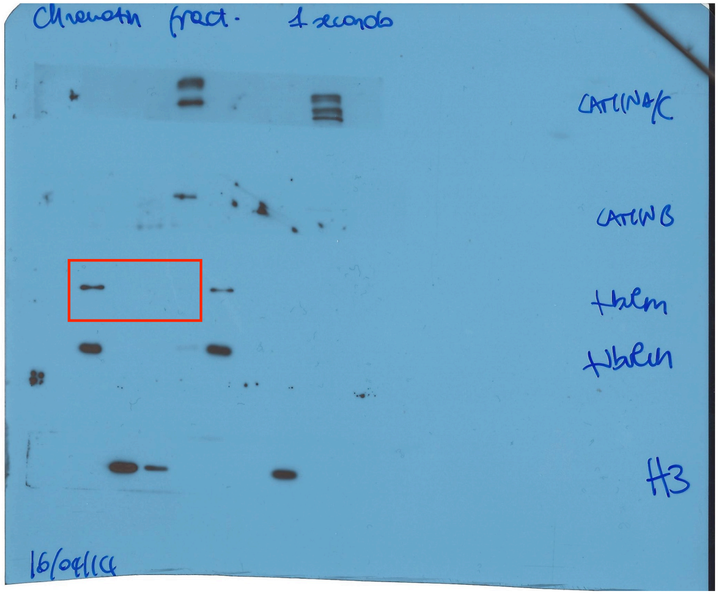

H3 shown in supplementary fig.1a

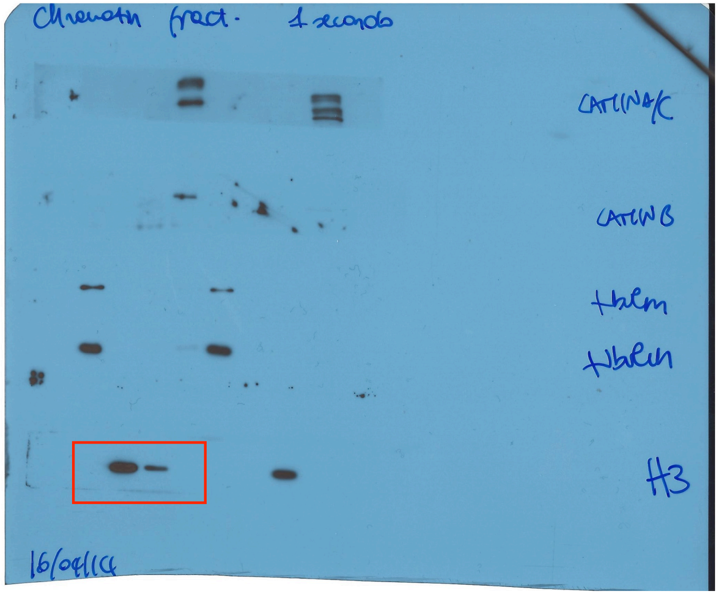

Lamin A/C shown in supplementary fig.1a

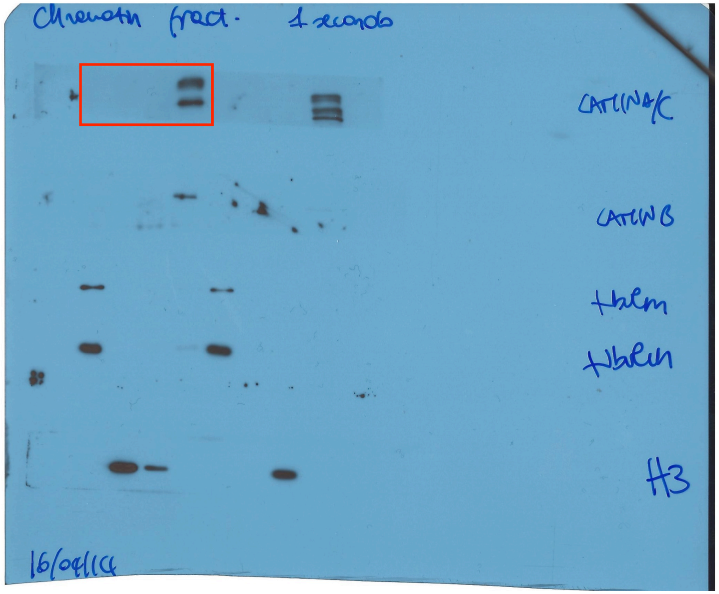

Lamin B shown in supplementary fig.1a

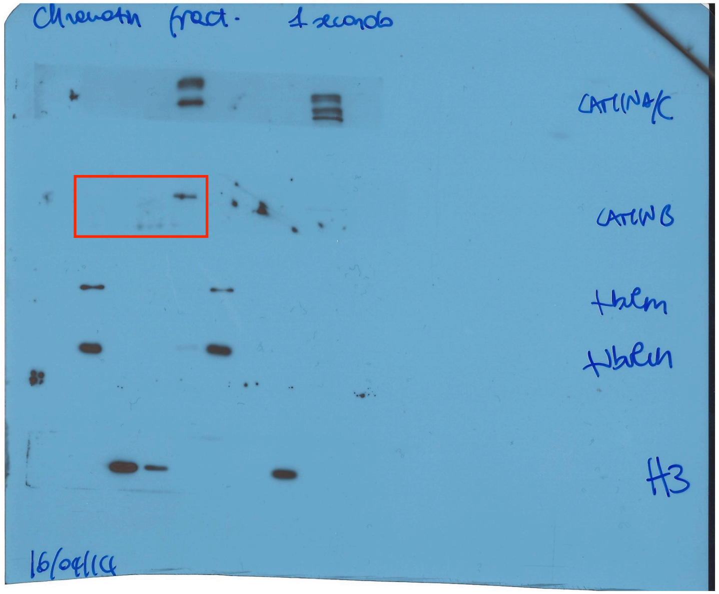

SUPPLEMENTARY FIGURE 4a

Actin shown in supplementary fig.4a

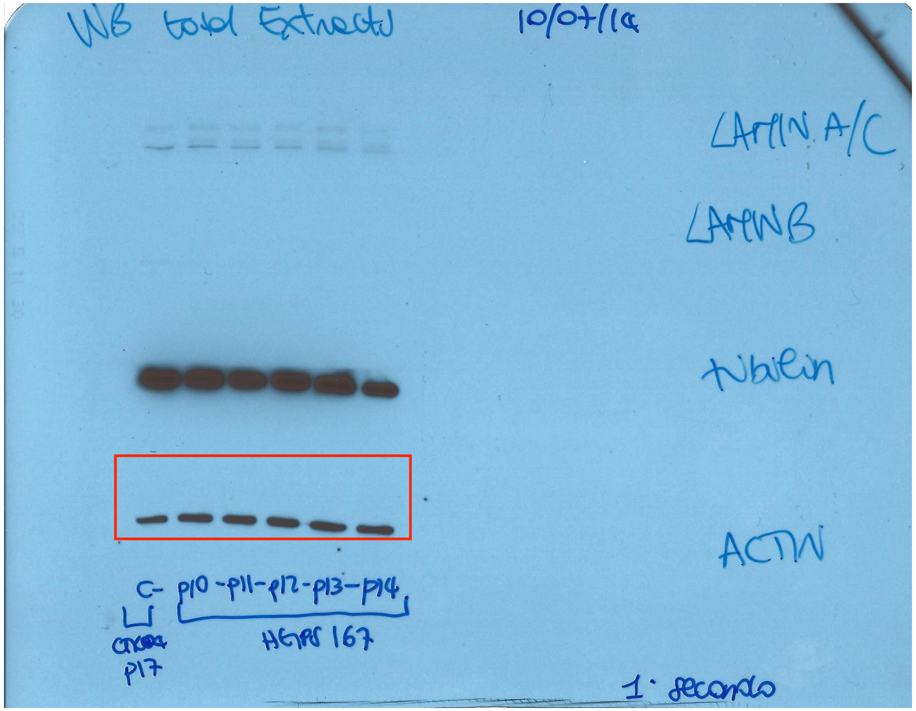

Lamin A/C shown in supplementary fig.4a

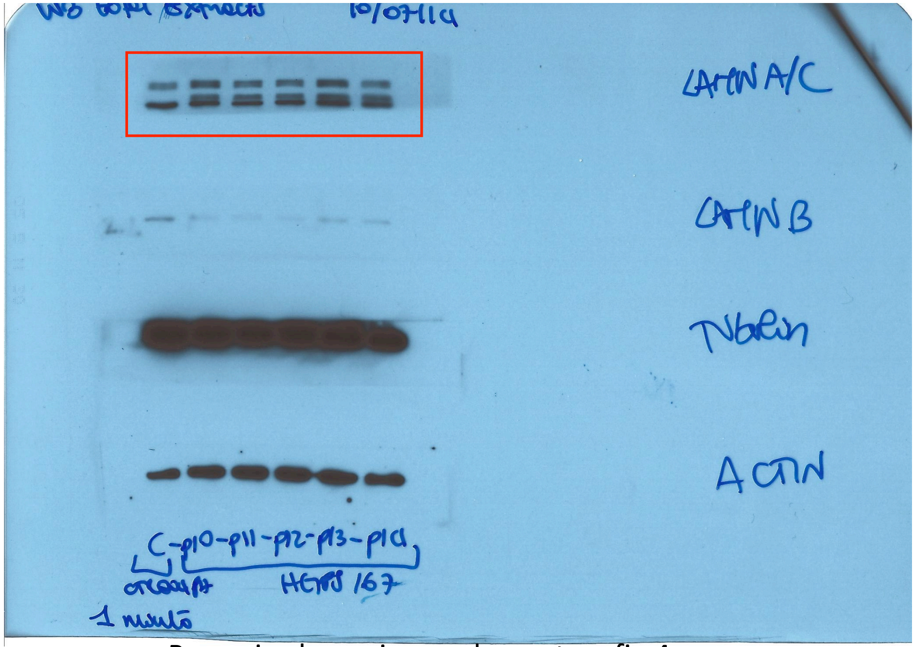

Lamin B shown in supplementary fig.4a

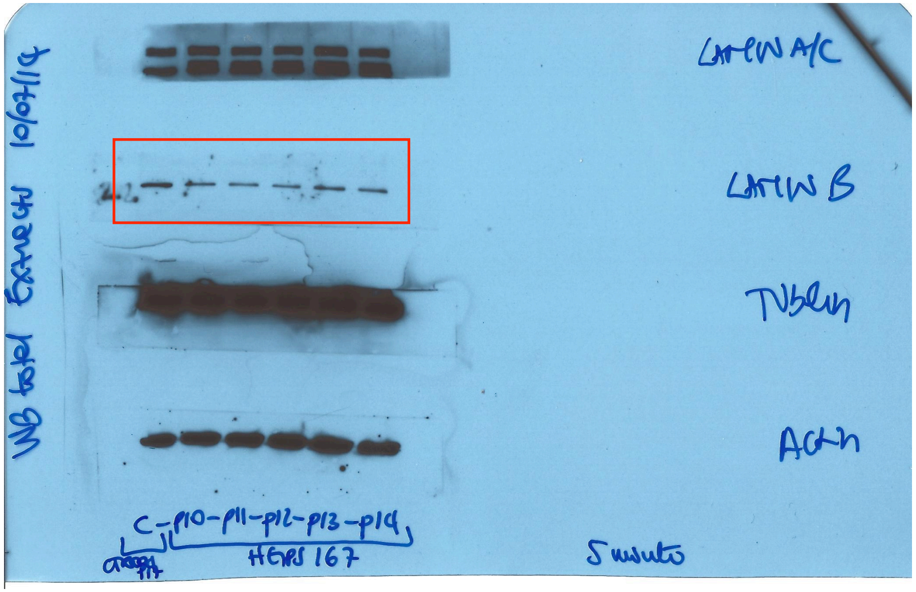

Progerin shown in supplementary fig.4a

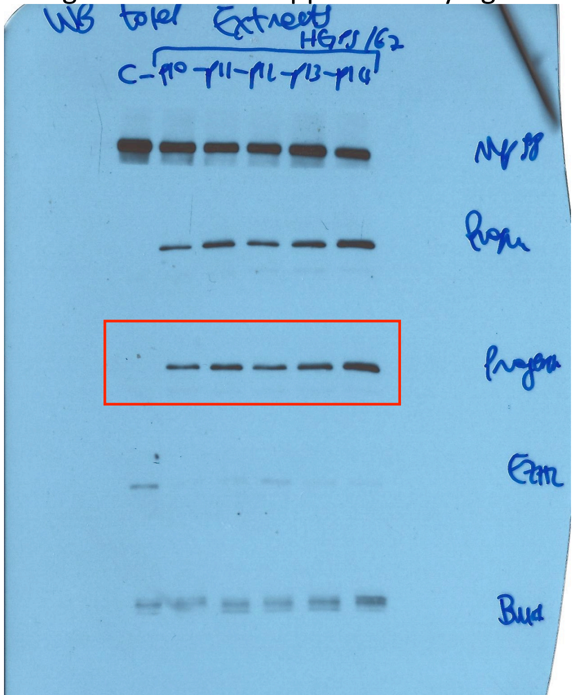

SUPPLEMENTARY FIGURE 6c

Lamin A/C shown in supplementary fig.6c

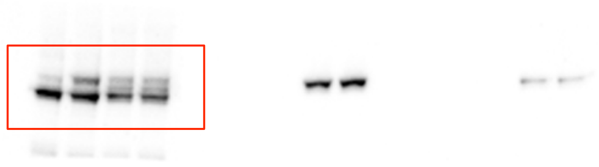

H3K9me3 shown in supplementary fig.6c

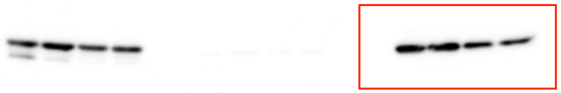

Progerin shown in supplementary fig.6c

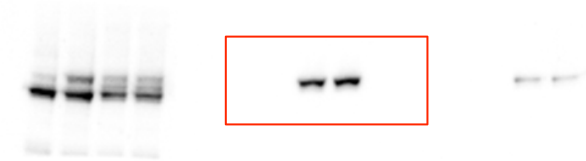

H3 low shown in supplementary fig.6c

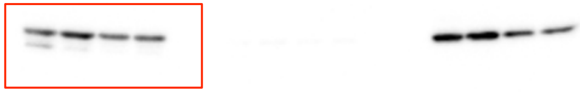

H3 high shown in supplementary fig.6c

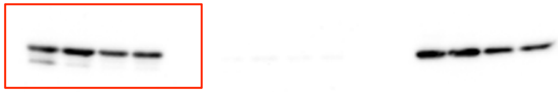

SUPPLEMENTARY FIGURE 8a

Ezh2 shown in supplementary fig.8a

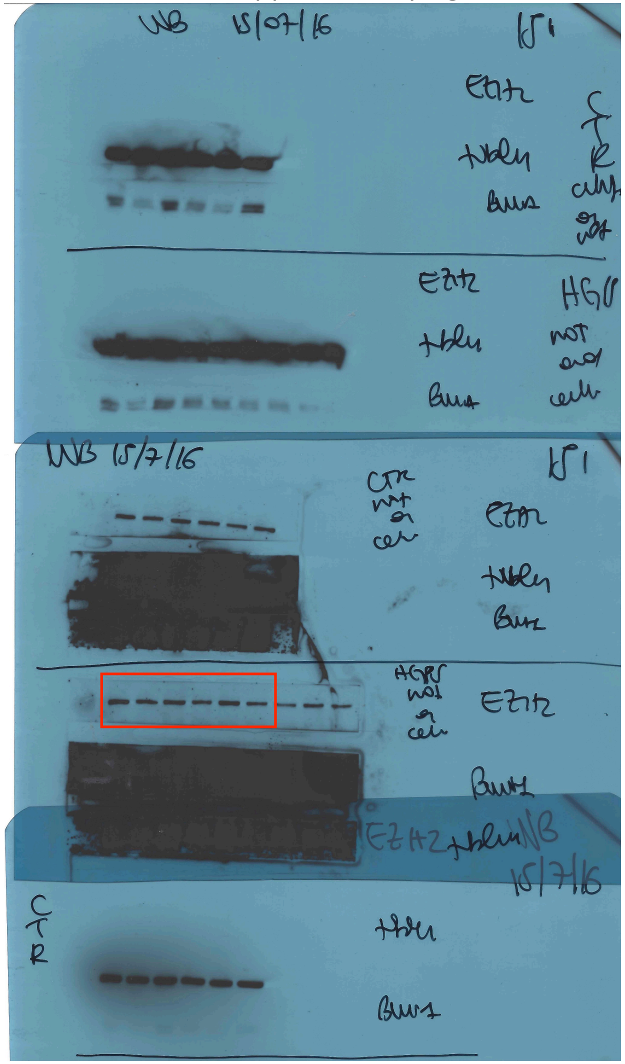

Bmi1 shown in supplementary fig.8a

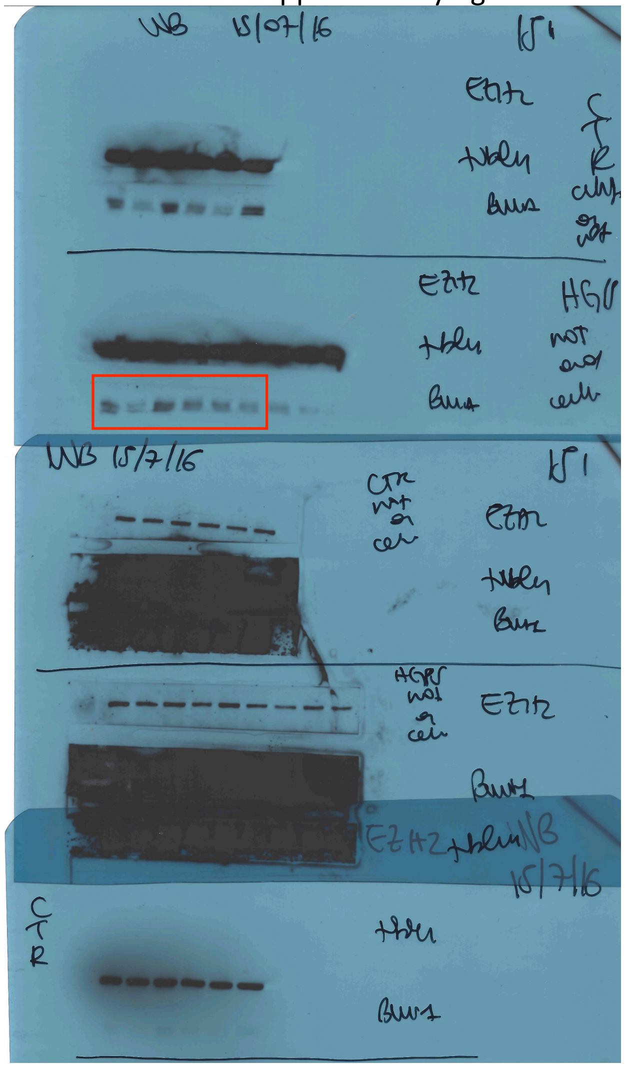

alfa-tubulin shown in supplementary fig.8a

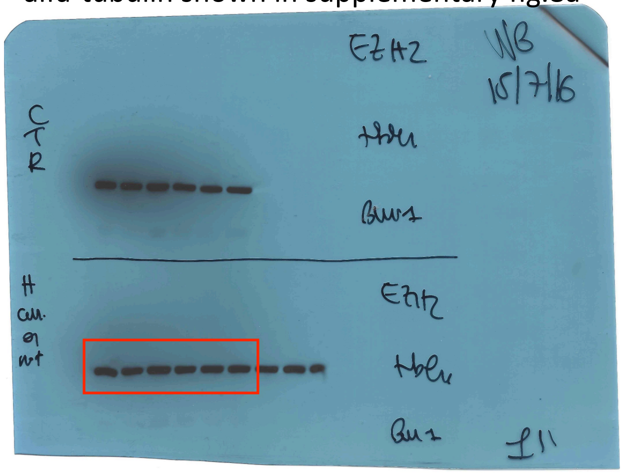

SUPPLEMENTARY FIGURE 8c

Lamin A/C shown in supplementary fig.8c

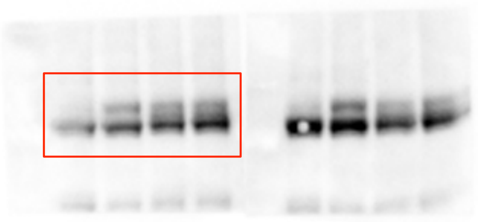

Progerin shown in supplementary fig.8c

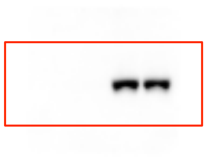

H3K27me3 shown in supplementary fig.8c

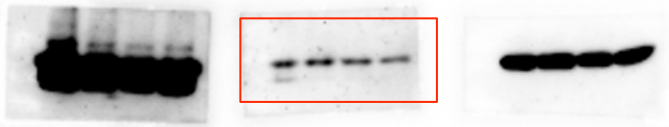

H3 shown in supplementary fig.8c

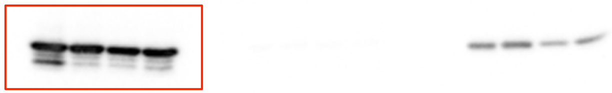

Supplement: Supplementary file 4 — Source Data [file 41467_2020_20048_MOESM4_ESM.zip › Uncropped Blots.pdf]
